# Individual contributions of exotoxins S and T on internalized *P. aeruginosa*

**DOI:** 10.1128/iai.00267-26

**Published:** 2026-06-15

**Authors:** Zachary J. Resko, Adam V. Thota, Christopher J. Corcoran, David G. Glanville, Seby Edassery, Keith A. Klein, Jonathan P. Allen, Jonathan A. Kirk, Andrew T. Ulijasz, Abby R. Kroken

**Affiliations:** 1 Department of Microbiology and Immunology, Loyola University Chicago 2456, Maywood, Illinois, USA; 2 Department of Medicine, Division of Pulmonary, Allergy, and Critical Care Medicine, University of Alabama Birmingham 9968, Birmingham, Alabama, USA; 3 Department of Cell and Molecular Physiology, Loyola University Chicago 2456, Maywood, Illinois, USA; University of California Davis, Davis, California, USA

**Keywords:** *Pseudomonas aeruginosa*, type three secretion system, host cell invasion, exoenzymes, intracellular pathogen, ADP-ribosylating toxins, epithelial cells

## Abstract

*Pseudomonas aeruginosa* is a high-priority pathogen and significant burden to health care worldwide. Although often regarded as an extracellular pathogen, *P. aeruginosa* is also capable of existing intracellularly in multiple cell types, including epithelial cells, goblet cells, and macrophages. This designation is attributed to two of its type three secretion system (T3SS)–dependent exotoxins, ExoS and ExoT, which inactivate host proteins that facilitate phagocytosis. However, studies investigating intracellular bacteria show that ExoS can paradoxically facilitate survival and replication, seemingly overriding the anti-internalization properties of itself and ExoT through its ADP-ribosylation activity. Here, we set out to define the individual and combined contributions of ExoS and ExoT by examining how each of their two functional domain activities affects epithelial cell invasion. Through *in vitro* biochemical assays, we found that ExoS is capable of ADP-ribosylating ExoT and their shared human cofactor, 14-3-3. We also found that ExoT ADP-ribosylates itself, unexpectedly enhancing its GTPase-activating protein (GAP) activity. Using fluorescence microscopy, we found that GAP activity of either exotoxin does not block internalization events, but is instead associated with fewer internalized bacteria entering a phase of rapid cytoplasmic replication. We demonstrate that ExoS ADP-ribosyltransferase activity is positively associated with vacuolar exit and cytoplasmic replication, and delivery of ExoS from extracellular bacteria triggers the intracellular T3SS⁻ subpopulation to become T3SS^+^ and exit the vacuole. Overall, this study highlights the underappreciated ability of *P. aeruginosa* to become intracellular and delineates how the enzymatic domains of ExoS and ExoT dictate the intracellular localization of the pathogen.

## INTRODUCTION

*Pseudomonas aeruginosa* is a ubiquitous opportunistic pathogen capable of infecting many human tissues. Prevalent sites of infection such as the skin, respiratory tract, and cornea may be made more susceptible through injury or inflammatory disease (1–5). While *P. aeruginosa* is an adept extracellular pathogen due to numerous secreted virulence factors, many studies over the last 30 years have reported mechanisms contributing to its ability to survive and replicate inside of multiple epithelial cell types (reviewed in [6]). A key component of *P. aeruginosa* virulence is its ability to deliver exotoxins directly to the host cell cytoplasm via a type three secretion system (T3SS) (7, 8). Previous studies have demonstrated that two exotoxins, ExoS and ExoT, have enzymatic functions that reduce the ability of *P. aeruginosa* to be internalized by host cells, while ExoS also promotes intracellular bacterial survival and replication (9–14).

ExoS and ExoT each consist of an N-terminal GTPase-activating (GAP) domain and a C-terminal adenosine diphosphate ribosyltransferase (ADPRT) domain (15, 16). GAP activity of ExoS, ExoT, and GAP effectors from other bacterial pathogens are widely regarded as antiphagocytic due to their ability to inhibit Cdc42, RhoA, and Rac, causing cytoskeletal destabilization (17–20). The ADPRT domains of ExoS and ExoT each transfer an ADP-ribose moiety derived from NAD^+^ to arginine residues, but they differ in their specific host targets. ExoS has been shown to ADP-ribosylate a broad range of host proteins, including Ras GTPases (21–26), vimentin (27), and adaptor proteins that link the cytoskeleton to the plasma membrane: ezrin, radixin, and moesin (ERM) (28). The repertoire of host targets of ExoT is limited to focal adhesion proteins, Crk1 and Crk2, and actin (11, 29). The catalytic activity of the ADPRT domain for both ExoS and ExoT depends on a conserved ExE biglutamic acid motif that facilitates NAD glycohydrolase and ADP-ribosyltransferase activity. Both effectors require host 14-3-3 proteins, which serve as chaperones for ADPRT activity (30, 31). Collectively, ADP-ribosylation of targeted host proteins compromises epithelial barrier integrity, enhancing *P. aeruginosa* infection.

Despite the role of GAP domains in preventing bacterial uptake, previous work has shown that the ADPRT domain of ExoS is paradoxically required to promote intracellular bacterial replication and survival (32). Infection of epithelial cells with effector-null *P. aeruginosa* mutants complemented with ExoS ADPRT drives the formation of membrane blebs, which serve as a niche for intracellular replication (32, 33). Further, ExoS ADPRT activity has been shown to delay lytic cell death in corneal epithelial cells through suppression of caspase-4-mediated pyroptosis (10). Alternatively, when only ExoT is expressed, bacteria appear to remain primarily extracellular (9, 13, 14). It remains unclear how the presence of ExoS negates the impact of ExoT to enhance internalization. One caveat with these prior studies is the use of multicopy complementation plasmids or full operon deletions, which have the potential to alter the stoichiometry of T3SS effectors delivered. Separate from the influence by T3SS effectors, the bacterial outer membrane lectin LecB has been shown to promote bacterial internalization (34). Host cell uptake pathways utilizing phosphoinositide 3-kinase (PI3K) are required for bacterial internalization (35), which is enhanced when the T3SS is locked off by mutating either its structural components (36) or the master transcription factor, ExsA (37). Furthermore, the expression of the T3SS is bistable, i.e., not all individual bacterial cells express it upon stimulus exposure (38, 39). This occurs because a subpopulation of bacterial cells are primed for rapid T3SS effector delivery through elevated cAMP, which transiently upregulates ExsA, leading to pre-assembled T3SS injectisomes (40, 41). It remains unknown whether bacteria are required to remain T3SS⁻ in order to enter host cells.

Early investigations of ExoS showed that it is capable of auto-ADP-ribosylation and self-inhibition of the GAP domain (42). In this work, purified ExoS GAP was found to incorporate [^32^P]NAD when incubated with purified ExoS ADPRT domain *in vitro*. Introduction of an R146K mutation in ExoS did not lead to complete loss of self-ADP-ribosylation, suggesting that other arginine residues may also be targeted. While not yet functionally investigated, ExoT is also thought to be capable of self-ADP-ribosylation (11, 29). In a recent study comparing purified ExoT ADPRT domain to full-length ExoT, self-ADP-ribosylation was only detected in the full-length protein, implying that ExoT can modify itself akin to ExoS (29).

In this study, we sought to clarify the functional relationship between the activities of ExoS and ExoT by interrogating the role each exotoxin plays in determining *P. aeruginosa* epithelial cell invasion, and whether self- or cross-ADP-ribosylation influences invasion. Using *in vitro* biochemical assays, we corroborate prior evidence of self-ADP-ribosylation of each exotoxin and also show that ExoS can ADP-ribosylate ExoT. ExoT, whether ADP-ribosylated by itself or by ExoS, retained its GAP activity. We also report 14-3-3 β as a substrate for ADP-ribosylation by ExoS and identify putative arginine residues of 14-3-3 β targeted by both ExoS and ExoT (29) using mass spectrometry. Contrary to the current paradigm, we found that the frequency of invasion by *P. aeruginosa* is not affected by the exotoxin domain expressed. Rather, expression of stand-alone GAP domains confines *P. aeruginosa* to intracellular vacuoles, whereas expression of ExoS ADPRT promotes vacuolar escape and cytoplasmic replication of this population in a T3SS-dependent manner. Further, we report evidence that delivery of ExoS ADPRT from extracellular bacteria can drive vacuolar exit of the intracellular subpopulation. Together, these results further our understanding of how the individual domain functions of the T3SS exotoxin facilitate *P. aeruginosa* intracellular survival and virulence.

## RESULTS

### ExoS and ExoT self- and cross-ADP-ribosylate *in vitro*

**Fig 1:**
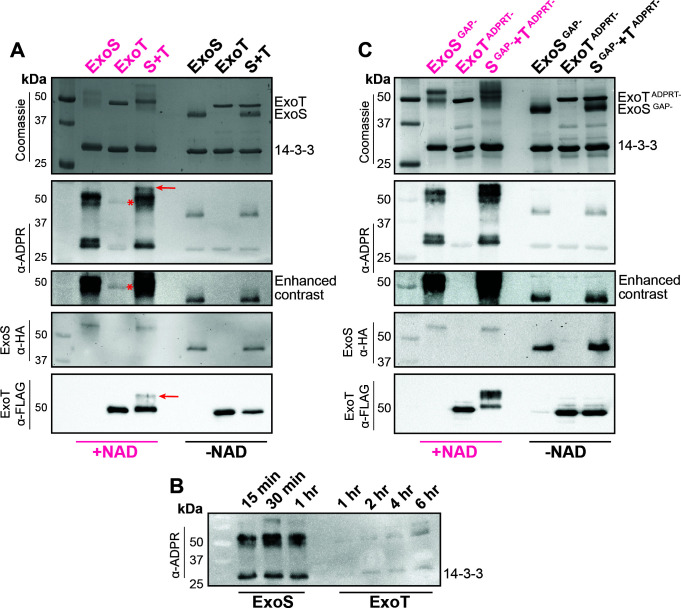
ExoT is self-ADP-ribosylated and cross-ADP-ribosylated by ExoS. (**A**) *In vitro* ADP-ribosylation assays were performed using 5 μg (Coomassie) or 2 μg (western blots) of each HA-ExoS and/or FLAG-ExoT and 14-3-3 β with or without the addition of NAD^+^ and incubated for 1 h at 25**°**C. Whole reactions were loaded onto the gels. Red asterisk indicates faint band showing auto-ADP-ribosylation of ExoT, which is also shown in a cropped blot for anti-ADP ribose. Red arrows indicate unique bands that occur only when ExoS and ExoT are co-incubated, which suggest cross-ADP-ribosylation. (**B**) Reactions to examine self-ADP-ribosylation kinetics were performed for the indicated times at 25**°**C. (**C**) The experiment was performed as in panel A, but using mutated ExoS lacking GAP activity (R146K) or ExoT lacking ADPRT activity (E383D, E385D). All images are representative of three replicate experiments.

Prior studies have demonstrated that ExoS is capable of self-ADP-ribosylation (42). To investigate potential self- and cross-modification activities between ExoS and ExoT, we purified recombinant proteins with both functional domains and assessed ADP-ribosylation biochemically using an *in vitro* assay. ExoS and ExoT were incubated with the purified cofactor, human 14-3-3 β, with or without the addition of NAD^+^ to allow for ADP-ribosylation, and modification was assessed by Western blots (Fig. 1A). While self-ADP-ribosylation was evident for both ExoS and ExoT, we detected a higher degree of modification for ExoS than ExoT (Fig. 1A). To compare the efficiency of self-ADP-ribosylation between ExoS and ExoT, we incubated *in vitro* reactions for increasing amounts of time. While ExoS is capable of modifying itself to near completion within 15 min in this assay, ExoT never fully reached similar levels of ADP-ribosylation but did accumulate over 6 h (Fig. 1B). We were intrigued to find evidence that 14-3-3 β is ADP-ribosylated by ExoS (Fig. 1A). Upon mixing ExoS and ExoT, we observed an additional high-molecular-weight band in both α-mono-ADPR and α-FLAG blots, which was absent in reactions containing either protein alone, suggesting the formation of a cross-ribosylated ExoT product (red arrows, Fig. 1A). These experiments were conducted at 25°C, as in previous reports. A shift of ExoT was also observed at 37°C (Fig. S1).

To directly demonstrate cross-ADP-ribosylation of ExoT by ExoS, we purified catalytic null versions of each protein to minimize the detection of interfering self-ADP-ribosylation. We mutated the catalytic arginine (R146K) of the ExoS GAP domain, believed to be the primary target of self-modification, and the biglutamic acid motif (E383D, E385D) of the ExoT ADPRT domain. *In vitro* ADP-ribosylation assays revealed that ExoS GAP⁻ ^R146K^ is still self-modified, highlighting that the catalytic GAP arginine is not the only amino acid targeted by self-ADP-ribosylation (Fig. 1C). ExoT ADPRT⁻ ^E383D, E385D^ ablated self-ADP-ribosylation, allowing us to investigate the ability of ExoS GAP⁻ ^R146K^ to ADP-ribosylate ExoT ADPRT⁻ ^E383D, E385D^. When incubated together, we observed enhanced detection of the shifted banding corresponding to ExoT in both α-mono-ADPR and α-FLAG blots (red arrows, Fig. 1C).

Together, these data show ADP-ribosylation capabilities of ExoS and ExoT on three fronts. First, ExoS and ExoT both undergo self-ADP-ribosylation *in vitro*, but ExoS appears to do so far more efficiently than ExoT under the conditions in this *in vitro* reaction. Second, we have identified the cofactor, 14-3-3 β, as a potential target of ADP-ribosylation that was previously unappreciated for ExoS. Lastly, we report that ExoS can ADP-ribosylate ExoT *in vitro*.

### ExoS ADP-ribosylation of ExoT does not alter GAP domain activity

**Fig 2:**
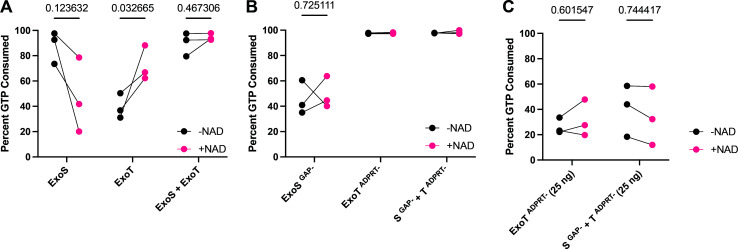
Effects of exotoxin GAP domain self-ADP-ribosylation and cross-ADP-ribosylation on RhoA GTPase activity. (**A**) First, *in vitro* ADP-ribosylation assays were performed using each exotoxin, with or without NAD^+^ supplementation, for 2 h. Following this, 100 ng of total exotoxin from the reactions was incubated with 100 ng of RhoA for 1 h, and GTP consumption was measured using a GTP-Glo Assay Kit. *P* values were calculated using Welch’s unpaired *t*-test, and exact values are reported. (**B**) The experiment was performed as in panel A, but using catalytically inactivated exotoxins as indicated. (**C**) The experiment was performed as in panel A, using only 25 ng of *in vitro* reaction exotoxins for incubation with 100 ng of RhoA.

Previous work suggests that *P. aeruginosa* strains expressing only ExoS had a higher tendency to invade epithelial cells than strains expressing only ExoT, which were thought to remain extracellular (9). If both toxins are present, invasion resembles that of a strain expressing only ExoS. We postulated that ExoS may ADP-ribosylate and inactivate the GAP domain of ExoT, effectively shutting down the antiphagocytic activity observed in strains expressing only ExoT. To measure the GAP activity of ADP-ribosylated effectors, we performed *in vitro* biochemical assays in which modified effectors were incubated with the GTPase human RhoA, and GTP consumption was measured. Surprisingly, self-ADP-ribosylation appeared to impact the GAP activity of ExoS and ExoT differently. ExoS exhibited trends of reduced GAP activity when self-ADP-ribosylated as previously shown (42), whereas ExoT GAP activity was increased significantly (Fig. 2A). Combining ExoS and ExoT led to sustained GAP activity regardless of the addition of NAD^+^ (Fig. 2A). To isolate and measure the influence of ExoS-mediated ADP-ribosylation on ExoT GAP activity, we performed GAP activity assays with the catalytic null versions of each exotoxin. As expected, ExoS GAP⁻ ^R146K^ had reduced GAP activity compared to WT ExoS and remained unchanged with NAD^+^ (Fig. 2B). ExoT ADPRT⁻ ^E383D, E385D^ appeared to have high specific activity when tested at the same concentration as WT ExoT, which limited our ability to interpret changes from NAD^+^ addition (Fig. 2B). We repeated assays with a reduced concentration of ExoS GAP⁻ ^R146K^ and ExoT ADPRT⁻ ^E383D, E385D^ to ensure detectable changes were within the linear range, observing no change in ExoT GAP activity between conditions with or without NAD^+^ (Fig. 2C). While our *in vitro* ADP-ribosylation assays suggest that ExoS is capable of cross-ADP-ribosylating ExoT (Fig. 1), this modification did not appear to influence the catalytic activity of the ExoT GAP domain.

### Identification of arginine targets for ADP-ribosylation in ExoS, ExoT, and 14-3-3 β

**Fig 3:**
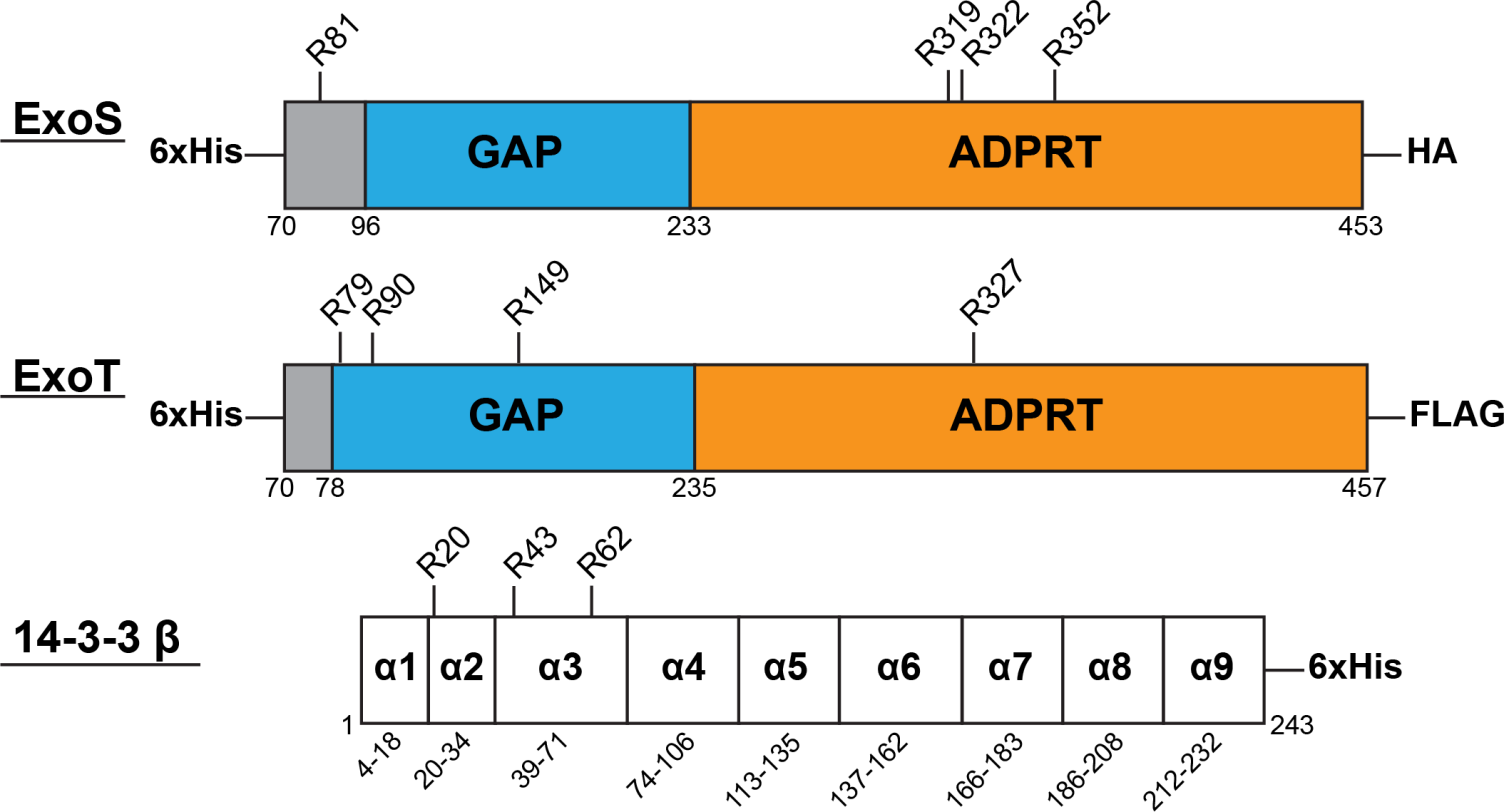
ADP-ribosylated arginine residues detected by mass spectrometry. Schematics of domain structures of all three recombinant purified proteins used in *in vitro* ADP-ribosylation assays. The ADP-ribosylated arginines detected by LC-MS/MS are annotated. No unique residues were identified specific to the cross-ADP-ribosylation reactions (see Table 1), and both ExoS and ExoT were able to ADP-ribosylate 14-3-3 β at all sites indicated.

Western blots probing for mono-ADP-ribosylation in our *in vitro* ADP-ribosylation assays show that the catalytic arginine, R146, in the GAP domain of ExoS is not the only arginine modified, and the cofactor, 14-3-3 β, may also be targeted for modification (Fig. 1). To identify other potential arginine targets for ADP-ribosylation in ExoS, ExoT, and 14-3-3 β, we performed liquid chromatography-tandem mass spectrometry (LC-MS/MS) on *in vitro* ADP-ribosylation reactions prepared as described above. From these reactions, we discovered multiple alternative arginine residues in both ExoS and ExoT that were ADP-ribosylated when NAD^+^ was supplemented (Table 1; Fig. 3). In ExoS, we detected R81, which is found within the unstructured domain preceding the GAP domain, as well as R319, R322, and R352, which, based on previously resolved crystal structures of ExoS ADPRT:14-3-3β:Carba-NAD, are all proximal to the predicted NAD^+^-binding pocket (31). Side chains of residues R319 and R322 are both oriented toward the NAD^+^-binding pocket of the ExoS ADPRT domain and are predicted to contribute to NAD^+^ binding. R352 is found within a predicted loop domain that changes conformation from *apo* to *holo* upon NAD^+^ binding, but is not predicted to bind NAD^+^ directly. Self-ADP-ribosylation of ExoS at R146 has not yet been detected directly using mass spectrometry, but inferred through reduced incorporation of radiolabeled NAD when R146 is mutated (42). While we were unable to detect ADP-ribosylation of the catalytic ExoS GAP R146, we did find modified ExoT GAP R149. Therefore, this modification is possible, but may occur at a low frequency in our reactions considering that the catalytic rate of the GAP domain increased (Fig. 2). We also found R327, an adjacent residue to the predicted NAD^+^-binding pocket of the ExoT ADPRT domain. Two arginine residues within the ExoT GAP domain, R79 and R90, were also identified. We did not identify any unique modified ExoT arginine residues when combined with ExoS, suggesting that cross-ADP-ribosylation may target the same arginine residues as ExoT self-modification, with ExoS exhibiting faster kinetics. In addition to the alternative arginine targets found in ExoS and ExoT, we also report three potential target arginine residues of 14-3-3 β: R20, R43, and R62. Of the 9 α-helix domains of 14-3-3 β, R20 is found within α2, and R43 and R62 are within α3.

**TABLE 1:** ADP-ribosylated arginine residues identified by mass spectrometry

*In vitro* reaction	ExoS	ExoT	14-3-3 β
ExoS + 14-3-3 β	R81 R319 R322 R352	N/A^*a*^	R20 R43 R62
ExoT + 14-3-3 β	N/A	R79 R90 R149 R327	R20 R43 R62
ExoS + ExoT + 14-3-3 β	R81 R319 R322 R352	R79 R90 R149 R327	R20 R43 R62

^*a*^ "N/A", not applicable.

### *P. aeruginosa* expressing only GAP domains retain invasion capabilities

**Fig 4:**
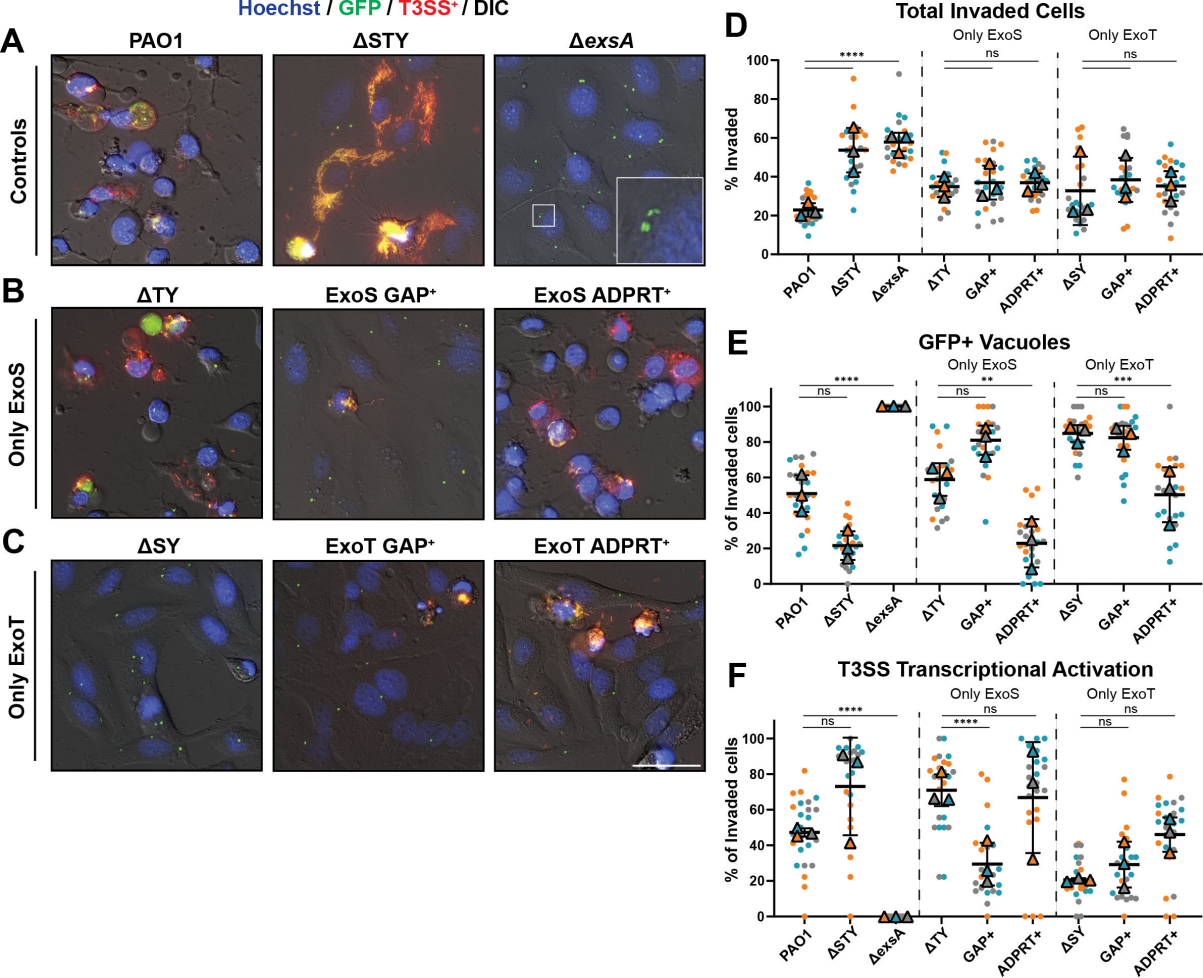
Expression of ExoS ADPRT leads to *P. aeruginosa* vacuolar escape, T3SS expression, and cytoplasmic replication. (**A–C**) HeLa cells were infected at an MOI of 10 for 3 h with either the indicated *P. aeruginosa* deletion mutants or genomic exotoxin catalytic null mutants expressing only the indicated active function domain of ExoS (**B**) or ExoT (**C**). At 3 h post-infection, amikacin (0.2 mg/mL) and polymyxin B (10 μg/mL) were supplemented, and cells were imaged every 30 min for 10 h. Representative images show infections at 8 h. The inset in the ∆*exsA* panel shows a zoomed view of individual bacteria. Scale bar = 50 µM. Movies S1 to S3 show full time-lapses from panels **A** to **C**. (**D**) The total percentage of infected cells were quantified from time-lapse movies. (**E**) The percentage of the invaded cells that contained GFP^+^ T3SS⁻ vacuolar bacteria was quantified. (**F**) The percentage of invaded cells containing GFP^+^ T3SS^+^ bacteria replicating cytosolically was quantified. For the quantification panels, data points from three independent biological replicates are shown and color-coded, with means (triangles) and standard deviations overlaid. Circles in a shared color signify individual microscopy fields within a biological replicate. Statistics were calculated using a non-parametric one-way ANOVA with Kruskal-Wallis multiple comparison test. **, *P* < 0.01; ***, *P* < 0.001; ****, *P* < 0.0001.

In previous studies, the effects of ExoS and ExoT GAP and ADPRT domains on epithelial cell invasion were investigated using mutants overexpressing each toxin and assessed for invasion based on either antibiotic exclusion or imaging of an *exoS* promoter-driven GFP (9, 14, 32). Thus, these studies leave open the possibility that internalized T3SS⁻ *P. aeruginosa*, which replicate slowly, remain underappreciated (9, 43). Here, we used a dual-reporter plasmid that allows us to track all *P. aeruginosa* using constitutively expressed sfGFP and visualize transcriptional regulation of the T3SS via *exoS* promoter-driven expression of mScarlet-I (44). To assess the contributions of each functional domain of ExoS and ExoT, we generated strains with chromosomal catalytic null point mutations in the GAP domain (ExoS^R146K^; ExoT^R149K^), ADPRT domain (ExoS^E379D, E381D^; ExoT^E383D, E385D^), or both for each exotoxin, and compared the percentage of HeLa cells containing intracellular bacteria among mutants (Fig. 4). All mutants were generated in an *exoY* ^K81M^catalytic null mutant background to isolate the effects of ExoS and ExoT, and secretion levels were determined to be similar by Western blot (Fig. S2) (36, 45). Off-target changes that could occur during sequential allelic exchange were examined using whole-genome sequencing of strains that had four catalytic activities nullified (Table S1). Offsite mutations occurred in phage proteins, hypothetical ORFs, and the DNA ligase LigA, none of which are anticipated to influence the activities examined here.

HeLa cells were infected at an MOI of 10 for 3 h, treated with amikacin and polymyxin B to both kill and reduce the fluorescence of extracellular bacteria through lysis, and internalized bacteria were then subsequently monitored by time-lapse microscopy from 4 to 14 h post-infection. Compared to WT PAO1 at 8 h, mutants lacking the T3SS effectors (Δ*exoSTY*) or the T3SS master regulator (Δ*exsA*) both occupied higher numbers of host cells but differed in their localization: the Δ*exoSTY* mutant quickly entered the cytoplasm and began replicating, while the Δ*exsA* mutant remained confined to the vacuolar compartment as T3SS⁻ GFP puncta, previously shown using transmission electron microscopy in a corneal epithelial cell line (Fig. 4A and D through F; Movie S1) (43, 46, 47). In strains expressing only WT or catalytic null versions of ExoS, we noted that expression of the ExoS ADPRT domain correlated with a higher tendency for *P. aeruginosa* to become T3SS^+^ and replicate in the cytoplasm compared to WT PAO1, whereas strains expressing active ExoS GAP were more frequently found in vacuoles (Fig. 4A versus Fig. 4B, E and F; Movie S2). Contrasting with results from prior studies suggesting that sole expression of ExoT prevents *P. aeruginosa* invasion (12–14, 48), we found that strains expressing WT ExoT or only active ExoT GAP could invade epithelial cells, but the bacteria were primarily confined to the vacuole with a lower frequency of T3SS activation compared to WT (Fig. 4C and D through F; Movie S3). Expression of only the active ExoT ADPRT domain increased the frequency of T3SS^+^ cytoplasmic replication relative to ExoT GAP expression (Fig. 4C and F), but was lower than that observed for strains expressing only an active ExoS ADPRT (Fig. 4B and F). Taken together, these data show that the GAP and ADPRT domains of ExoS and ExoT do not affect the overall frequency of host cell invasion (Fig. 4D); rather, each functional domain appears to influence the ability of *P. aeruginosa* to escape its initial subcellular compartment following invasion to replicate within the cytoplasm.

### ExoS ADPRT activity plays a key role in driving vacuolar exit

**Fig 5:**
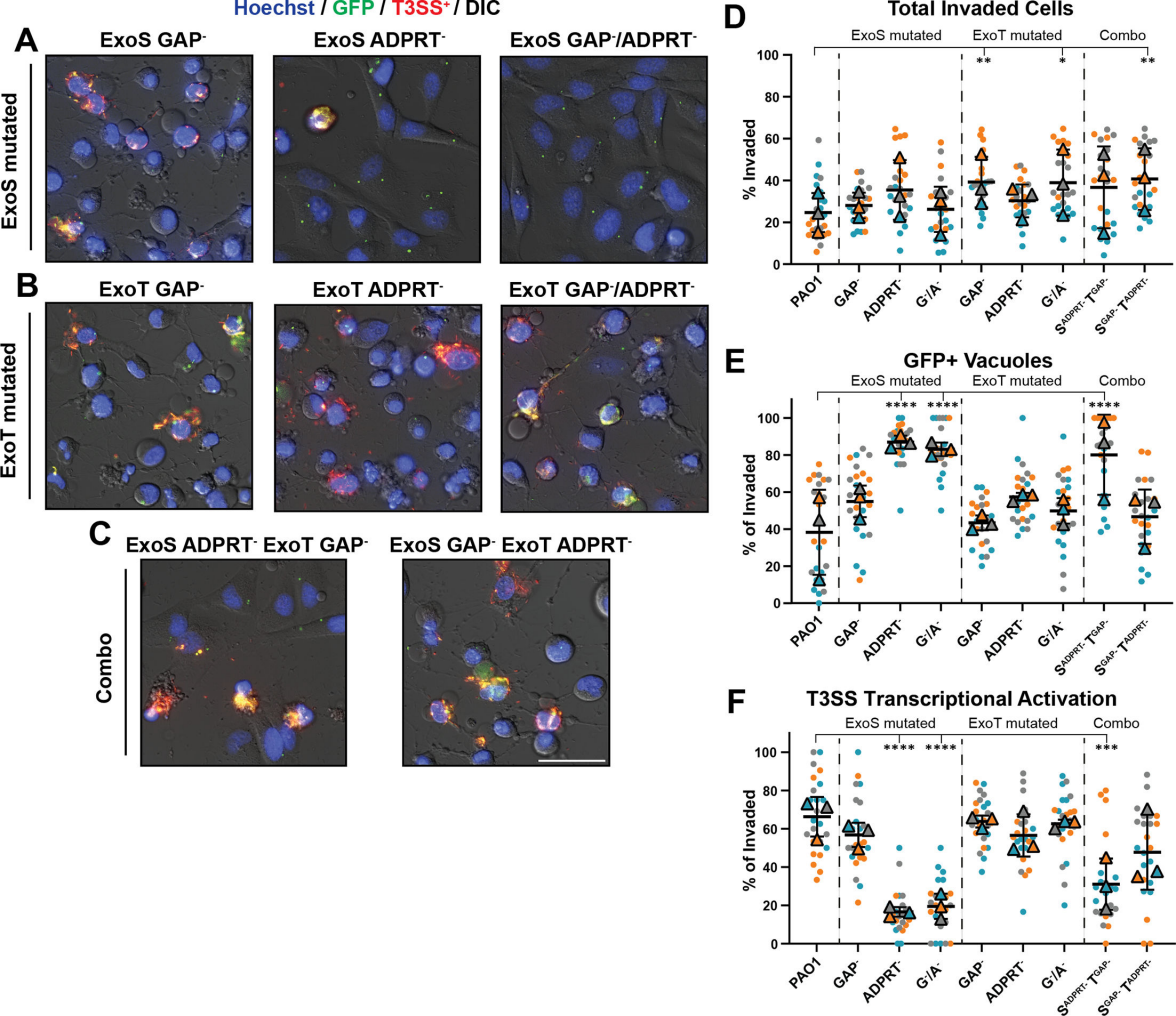
Catalytic inactivation of ExoS ADPRT disrupts *P. aeruginosa* vacuolar escape. (**A–C**) HeLa cells were infected at an MOI of 10 for 3 h with *P. aeruginosa* strains expressing indicated genomic catalytic null mutations of ExoS or ExoT, or both. At 3 h post-infection, amikacin (0.2 mg/mL) and polymyxin B (10 µg/mL) were supplemented, and cells were imaged every 30 min for 10 h. Representative images show infections at 8 h. Scale bar = 50 µM. Movies S4 to S6 show full time-lapses from panels **A** to **C**. (**D**) The total percentage of infected cells was quantified from time-lapse movies. (**E**) The percentage of the invaded cells that contained GFP^+^ T3SS⁻ vacuolar bacteria was quantified. (**F**) The percentage of the invaded cells that contained GFP^+^ T3SS^+^ replicating cytosolically was quantified. Data points from three independent biological replicates (circles) are shown and color-coded, with means (triangles) and standard deviations overlaid. Statistics were calculated using a non-parametric one-way ANOVA with Kruskal-Wallis multiple comparison test. *, *P* < 0.05; **, *P* < 0.01; ***, *P* < 0.001; ****, *P* < 0.0001.

While epithelial cell infections with strains individually expressing single active domains of ExoS or ExoT demonstrated how each influences intracellular localization, we sought to determine how this localization may change when combinations of active GAP and ADPRT domains are expressed together. Thus, we generated strains with chromosomal catalytic null mutants inactivating either one or both domains of ExoS or ExoT at a time and measured the frequency of epithelial cell invasion and T3SS transcriptional activity using the dual-reporter plasmid (Fig. 5). For strains expressing catalytic null versions of ExoS, we found that inactivation of the ExoS ADPRT led to an increased frequency of T3SS⁻ vacuolar *P. aeruginosa* (Fig. 5A and E) and a concomitant decrease in T3SS^+^ cytoplasmic replication (Fig. 5A and F; Movie S4). Catalytic inactivation of only the ExoT GAP and ADPRT domains did not significantly affect intracellular localization relative to WT (Fig. 5B and E and F; Movie S5).

To test how single domains of ExoS and ExoT may impact each other’s functions during host cell invasion, we also made catalytic null mutants by inactivating one domain of both ExoS and ExoT (Fig. 5C; Movie S6). We found that inactivation of the ExoT GAP domain and ExoS ADPRT domain in the same strain caused an observable increase in the frequency of T3SS^+^ *P. aeruginosa* replicating in the cytoplasm (Fig. 5C and F) when compared to strains in which only the ExoS ADPRT domain is mutated. Of note, when ExoS ADPRT activity was paired with ExoT GAP activity (which it cannot inactivate, see Fig. 2), there was no observable difference in host cell internalization rates versus when it was paired with ExoS GAP (Fig. 5D, compare column 7 to 9). In sum, the presence of the ExoS ADPRT activity enhances the probability of vacuolar exit and cytoplasmic replication independently from GAP domain activity.

### Extracellular delivery of ExoS ADPRT facilitates intracellular vacuolar escape

Our observations from Fig. 4 and 5 indicated that strains with catalytically inactivated ExoS ADPRT are generally restricted to vacuoles in a T3SS⁻ state and, thus, are not delivering exotoxins to the cytoplasm from the vacuole locale. This remains true even if other effector activities are present (Fig. 5). Because the effector-null strain, ∆*exoSTY*, can also exit vacuoles (Fig. 4A; Movie S1), it was difficult to envision how the effector repertoire influences vacuole exit. We reasoned that the effectors influencing the T3SS⁻ subpopulation are present but must be delivered by extracellular bacteria during our infections. This hypothesis aligns with the well-known phenomenon that effectors can elicit negative feedback; e.g., T3SS⁻ bacteria contacting a previously T3SS-intoxicated cell remain T3SS⁻ (49). A recent publication from Schator et al. arrived at a similar conclusion, in which extracellular *P. aeruginosa* was shown to facilitate the vacuolar escape of intracellular bacteria (50).

**Fig 6:**
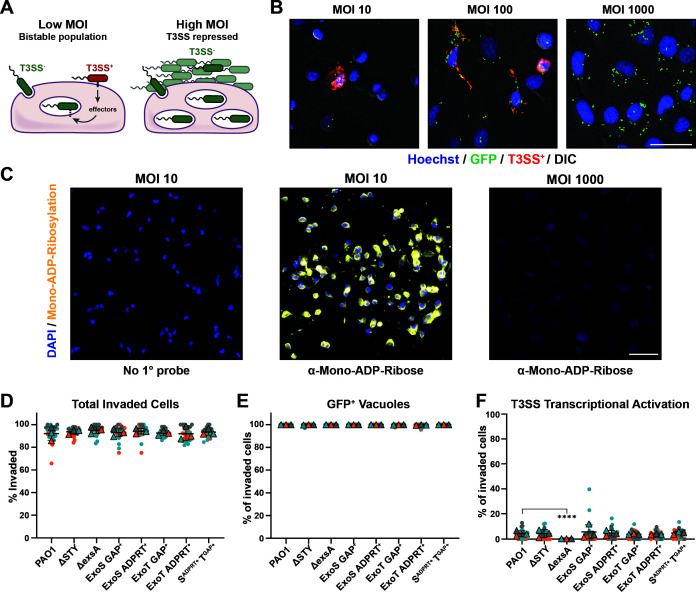
*P. aeruginosa* infections with an MOI of 1,000 repress extracellular T3SS activation. (**A**) Schematic depicting the tendency for T3SS activation of extracellular bacteria during epithelial cell infection with a low inoculum (MOI 10) and high inoculum (MOI 1,000). (**B**) HeLa cells were infected with PAO1 at an MOI of 10, 100, or 1,000 for 3 h, treated with amikacin (0.2 mg/mL) and polymyxin B (10 μg/mL), and imaged every 30 min for 14 h. Representative images show infections at 8 h. Scale bar = 50 µM. Movie S7 shows full time-lapses of images from panel **B**. (**C**) Immunofluorescent staining using a probe for mono-ADP-ribosylation was performed on similar infections as in panel B to detect T3SS effector delivery at 4 h post-infection. Scale bar = 50 µM. (**D–F**) HeLa cells were infected with exotoxin catalytic null mutant strains at an MOI of 1,000 for 2 h, treated with antibiotics as described in panel **A**, and imaged every 30 min for 14 h. The total percentage of invaded cells was quantified from time-lapse movies (**D**), as well as the percentage of the invaded cells that contained GFP^+^ T3SS⁻ vacuolar bacteria (panel E) and GFP^+^ T3SS^+^ replicating cytosolically (**F**). Data points from three independent biological replicates (circles) are shown and color-coded, with means (triangles) and standard deviations overlaid. Statistics were calculated using a non-parametric one-way ANOVA with Kruskal-Wallis multiple comparison test. ****, *P* < 0.0001.

Thus far, our data indicate that ExoS ADPRT activity is a key component of vacuolar escape, but that it may come from extracellular bacteria. To test this, we first sought to eliminate the contributions of extracellular bacterial secretion and then track the capacity of the intracellular vacuolar subpopulation of *P. aeruginosa* to escape and replicate within the cytoplasm. To do this, we infected HeLa cells with a more concentrated inoculum (MOI 1,000), which we serendipitously discovered suppresses T3SS activation to near completion (Fig. 6A and B). As we increased the MOI, we observed a decrease in HeLa cell rounding, indicative of a decrease in T3SS effector delivery, as well as an increase in overall quantity of T3SS⁻ vacuolar bacteria per cell (Fig. 6B; Movie S7). To validate that increasing inoculum concentration mitigated external T3SS effector intoxication, we performed immunofluorescent staining for mono-ADP-ribosylation on PAO1-infected HeLa cells at an MOI of 10 or 1,000 and found that ADP-ribosylation in cells infected at an MOI of 1,000 was, strikingly, almost completely eliminated (Fig. 6C). In agreement with host cells remaining motile and retaining normal morphology, cell survival was preserved at the high inoculum (Fig. S3). By mitigating the influence of T3SS effectors delivered extracellularly, we were able to monitor the ability of vacuolar *P. aeruginosa* to escape and replicate in the host cytoplasm over time without the influence of externally delivered T3SS effectors. We found that infection of HeLa cells at an MOI of 1,000 led to a large increase in the percentage of cells with intracellular bacteria (Fig. 6D) compared to an MOI of 10 (Fig. 4D and 5D), where, at an MOI of 1,000, effectively every invaded cell had multiple vacuolar GFP^+^ *P. aeruginosa* populations (Fig. 6E) that seldom escape for T3SS^+^ cytoplasmic replication over 18 h post-infection (Fig. 6F). Thus, it appears that the presence of a T3SS^+^ extracellular population of *P. aeruginosa* modulates the T3SS⁻ intracellular populations' ability to escape the vacuole and replicate in the host cell cytoplasm, in agreement with Schator et al. (50).

**Fig 7:**
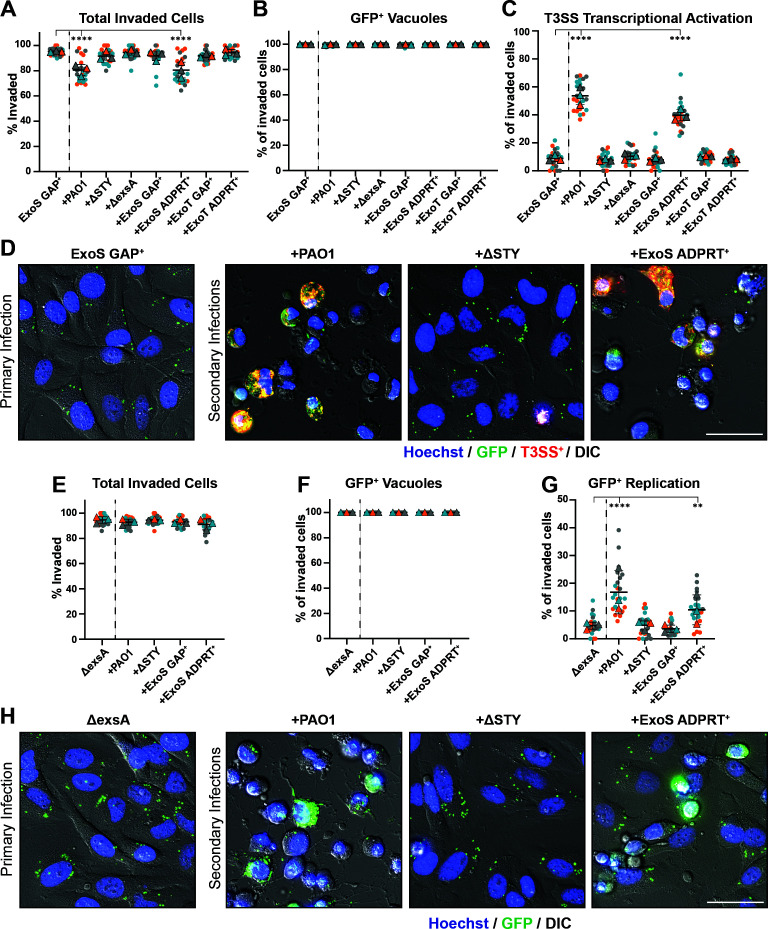
Extracellular delivery of ExoS ADPRT promotes intracellular vacuole escape. (**A–C**) HeLa cells were infected with an ExoS GAP^+^-expressing strain transformed with the fluorescent T3SS reporter plasmid at an MOI of 1,000 for 2 h, treated with amikacin (0.2 mg/mL) and polymyxin B (10 μg/mL) for 1 h, and then sequentially infected with the indicated non-fluorescent mutant strain expressing a single active ExoS or ExoT domain at an MOI of 10 for 3 h. Infected cells were treated again with antibiotics as described above and imaged every 30 min for 14 h. The total percentage of infected cells was quantified from time-lapse movies in panel **A**. The percentage of the invaded cells that contained GFP^+^ T3SS⁻ vacuolar bacteria and GFP^+^ T3SS^+^ replicating cytoplasmically were plotted in panels B and C, respectively, as a percentage of total invaded cells. (**D**) Representative images of infections from panels **A to C** between 8 and 10 h post-infection. Scale bar = 50 µM. Movie S8 shows full time-lapses. (**E–G**) Experiments were performed as in panel **A**; however, cells were initially loaded with Δ*exsA* mutant bacteria prior to the indicated sequential infection. Here, the frequency of GFP^+^ replication was quantified in panel **G**, which was visually identified through increasing areas of GFP^+^ bacteria confined within a cell, as this mutant is incapable of T3SS activation. Data points from three independent biological replicates (circles) are shown and color-coded, with means (triangles) and standard deviations overlaid. Statistics were calculated using a non-parametric one-way ANOVA with Kruskal-Wallis multiple comparison test. **, *P* < 0.01; ****, *P* < 0.0001. (**H**) Representative images of infections from panels **E to G** between 8 and 10 h post-infection. Scale bar = 50 µM. Movie S9 shows full time-lapses.

After an MOI of 1,000 was determined ideal for our experiments to interrogate the influence of extracellular effectors on the T3SS⁻ vacuolar population, we proceeded to infect HeLa cells with (MOI of 1,000) using an ExoS GAP^+^-expressing strain (that harbored the dual color T3SS reporter) which predominantly localizes to vacuoles. After 2 h, we eliminated the remaining extracellular bacteria with antibiotic treatment and subsequently infected cells again with a non-fluorescent strain expressing a single active functional domain of ExoS or ExoT at an MOI of 10 (Fig. 7A through D). Similar to what we observed in Fig. 4 and 5, we found that secondary infections with a strain externally delivering active ExoS ADPRT (PAO1 and ExoS ADPRT^+^) led to vacuolar escape corresponding with a switch to T3SS^+^ and cytoplasmic replication of the fluorescent ExoS GAP^+^ *P. aeruginosa* (Fig. 7A through D; Movie S8). Secondary infection with the Δ*exoSTY* mutant, which still produces the T3SS apparatus but lacks effectors, did not lead to vacuolar escape, suggesting that this phenomenon may be attributed to the host cell manipulation by ExoS ADPRT.

### Production of the T3SS apparatus is important for optimal vacuolar escape

Our data indicate that extracellular delivery of active ExoS ADPRT influences the T3SS⁻ intracellular population to escape the vacuole and replicate in a T3SS^+^ state. However, we needed to understand if this was (i) a result of the externally delivered ExoS ADPRT influencing the vacuolar population’s bistability state (6, 39), (ii) vacuolar destabilization through disruption of host cell cytoskeletal elements by ExoS ADPRT (27, 28, 51–53), or (iii) both. To determine if extracellular delivery of active ExoS ADPRT is sufficient to promote vacuolar escape, we performed sequential infections in which HeLa cells were first infected with a fluorescent Δ*exsA* (T3SS⁻) mutant at an MOI of 1,000 for 2 h and then secondarily infected with non-fluorescent *P. aeruginosa* strains expressing a single active effector domain at an MOI of 10 (Fig. 7E through H). While a large majority of HeLa cells harbored intracellular vacuolar Δ*exsA P. aeruginosa* (Fig. 7E and F; Movie S9), secondary infection with ExoS ADPRT^+^-expressing strains only partially facilitated vacuolar escape and cytoplasmic replication of this T3SS⁻ strain (Fig. 7G)—approximately 30% of what we quantified for a T3SS-competent strain (Fig. 7C, note reduced Y-axis scale). Together, these data suggest that extracellularly delivered ExoS ADPRT can partially mediate the vacuolar escape of intracellular *P. aeruginosa* populations through a host cell target-driven mechanism, but may also influence the T3SS bistability state of vacuolar populations to promote maximum vacuolar exit.

### Effectors influence bacterial trafficking in primary corneal epithelial cells

**Fig 8:**
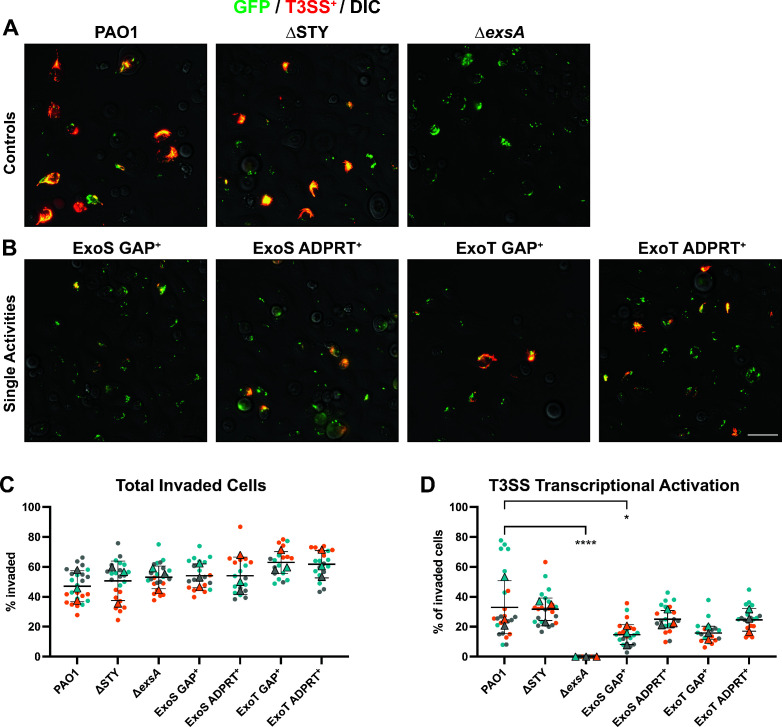
Exotoxin domains influence bacterial subcellular niches in primary corneal epithelial cells. (**A**) Primary human corneal epithelial cells were infected with an MOI of 10 for 3 h with WT PAO1 or the indicated *P. aeruginosa* deletion mutants. At 3 h post-infection, amikacin (0.2 mg/mL) and polymyxin B (10 μg/mL) were supplemented, and cells were imaged every 30 min for 10 h. Representative images show infections at 8 h. Scale bar = 50 µM. Movie S10 shows full time-lapses from panels **A** and **B**. (**B**) Experiments were performed as in panel A, but using *P. aeruginosa* catalytic point mutants expressing only the remaining activities indicated. Movie S10 shows full time-lapses from panels A and B. (**C**) The total percentage of infected cells was quantified from time-lapse movies. (**D**) The percentage of the invaded cells that contained GFP^+^ T3SS^+^ replicating cytosolically. Data points from three independent biological replicates (circles) are shown and color-coded, with means (triangles) and standard deviations overlaid. Statistics were calculated using a non-parametric one-way ANOVA with Kruskal-Wallis multiple comparison test. *, *P* < 0.05; ****, *P* < 0.0001.

HeLa cells have long served as a useful reductionist system for bacterial toxin studies, but can fail to recapitulate important host epithelial biology due to transformation and chromosomal instability. To this end, we also tested our key findings in primary human corneal epithelial cells, since incidents of intracellular *P. aeruginosa* are well established in corneal cell lines and mouse cornea infection models (33, 50, 54). Bacteria encoding single-effector activities showed a similar trend to that observed in HeLa cells: WT PAO1 and PAO1∆*exoSTY* showed populations of T3SS^+^ cytoplasmic replication, whereas ∆*exsA* mutants were generally confined to vacuoles (Fig. 8A). Nearly 100% of invaded cells contained at least some T3SS⁻ bacteria, so this was not plotted. When examining single-effector activity mutants, GAP^+^ strains tended to remain confined to T3SS⁻ puncta, although only ExoS GAP^+^ bacteria achieved statistical significance relative to WT PAO1 (Fig. 8B through D). Here, ExoT ADPRT provided a similar advantage as ExoS ADPRT in supporting the presence of cytoplasmic T3SS^+^ populations. We noted that spurious T3SS^+^ cytoplasmic exit occurred more frequently than expected when compared to HeLa cells, and that invaded primary cells appeared to die earlier in the time-lapse period examined in these experiments (Movie S9). Early host cell death could be the result of functional inflammasome pathways in corneal cells that are missing in HeLa cells (10). Cell population heterogeneity could also contribute to differences from HeLa cells; cell size was highly variable, and cells reaching terminal senescence may respond to pathogens differently.

## DISCUSSION

**Fig 9:**
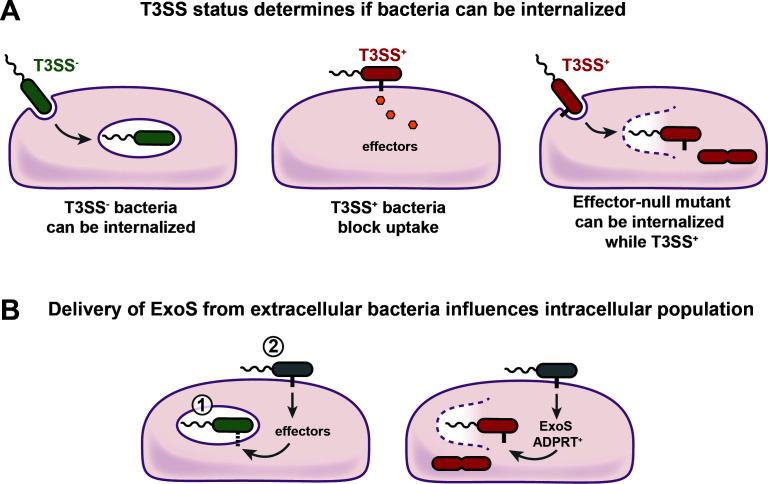
Model showing how T3SS status and ExoS delivery determine the subcellular locations of bacteria. (**A**) T3SS status governs the initial invasion capability of an individual bacterium. When T3SS^+^ bacteria are internalized due to deletion of the effectors, they efficiently access the host cell cytoplasm. (**B**) The T3SS state of vacuolar bacteria is positively influenced by ExoS when it is delivered by extracellular bacteria, which promotes vacuole exit and replication in the cytoplasm.

In this study, we sought to characterize the functional relationship between the *P. aeruginosa* exotoxins, ExoS and ExoT, and delineate the role each exotoxin plays in dictating epithelial cell invasion capabilities. Through *in vitro* biochemical assays, we not only found that ExoT is capable of self-ADP-ribosylation, but also has the potential for cross-ADP-ribosylation by ExoS. Neither of these modifications appears to inhibit the activity of the ExoT GAP domain. Additionally, we report that the host cofactor for exotoxin ADPRT activity, 14-3-3 β, is a target substrate for ExoS and ExoT ADP-ribosylation. Using time-lapse fluorescent microscopy, we demonstrated that catalytic inactivation of the ExoS and ExoT GAP and/or ADPRT domains does not affect invasion frequency (Fig. 9A). Rather, the ability of *P. aeruginosa* to escape the vacuole and replicate within the cytoplasm is primarily dependent on the activity of the ExoS ADPRT domain. In addition, we discovered that delivery of ExoS ADPRT from the T3SS^+^ extracellular subpopulation can promote the vacuolar escape of the T3SS⁻ intracellular subpopulation through potential modulation of both host cell processes and intracellular bacterial T3SS activation (Fig. 9B).

Prior investigations into the functions of ExoS and ExoT during epithelial cell invasion generally linked ExoS ADPRT activity to promoting invasion (32, 33), while ExoT GAP activity prevented bacterial uptake (9, 12, 13). Given that *P. aeruginosa* expressing both exotoxins is still able to invade epithelial cells, we postulated that the ExoT GAP domain may be inactivated through ADP-ribosylation by either ExoS or itself. *In vitro* ADP-ribosylation assays using purified recombinant exotoxins demonstrated that both are possible (Fig. 1), suggesting that interplay between ExoS and ExoT self- and cross-ADP-ribosylation during infection may be a mechanism by which *P. aeruginosa* regulates exotoxin activity. While our data support the notion that ExoT is capable of self-ADP-ribosylation akin to ExoS, it seemingly occurs less efficiently when compared to ExoS (Fig. 1B), indicating that ExoT self-ADP-ribosylation of the GAP domain is not a predominant regulatory mechanism compatible with the timing events of early infection. Previous ExoS and ExoT enzyme kinetic studies support this concept, in which ExoT was described to have about 0.2% of the specific activity of ExoS and a lower velocity rate in ADP-ribosylating soybean trypsin inhibitor (SBTI), despite similar binding affinities for NAD^+^ and SBTI (55). In biochemical assays measuring GAP domain activity on RhoA GTPase, we found that the addition of NAD^+^ to allow for ADP-ribosylation had different effects on ExoS and ExoT GAP domains: ExoS GAP activity was decreased upon self-ADP-ribosylation, whereas ExoT GAP activity was increased (Fig. 2A). Supporting this, catalytic inactivation of the ExoT ADPRT domain ablated the increase in RhoA GTP consumption upon NAD^+^ addition in conditions with ExoT ADPRT⁻ ^E383D, E385D^alone (Fig. 2C). Using mass spectrometry, we identified a few previously unknown ADP-ribosylated arginine residues in ExoT (R79 & R90) located in the ExoT GAP domain. In future studies, we plan to examine the regulatory effects of ADP-ribosylation at these residues and explore if and when these modifications of ExoS and ExoT are deployed during *in vivo* infection.

14-3-3 is a ubiquitous and highly conserved protein in eukaryotic tissues, known to interact with over 200 different proteins including kinases, phosphatases, and receptors, in a phosphorylation-dependent manner (56, 57). These interactions influence a multitude of cellular signaling pathways controlling cell survival, stress responses, and apoptosis (58). Prior studies have demonstrated that post-translational modifications, such as Ser/Thr/Tyr phosphorylation, fatty acylation, acetylation, and polyglycylation, can modulate 14-3-3 interactions (reviewed in reference 59); here, we report the first evidence substantiating that 14-3-3 β may also be mono-ADP-ribosylated by *P. aeruginosa* exotoxins (Fig. 1 and 3; Table 1). The physiological impact of 14-3-3 ADP-ribosylation is currently unknown. However, we speculate that the detected ADP-ribosylated arginines R20, R43, and R62, located in helices α2 and α3 of 14-3-3, could influence oligomerization, as helices α1–α4 are known to participate in dimer formation (60), or potentially disrupt interactions with target host signaling proteins. Future experiments interrogating the occurrence of these modifications to 14-3-3 β and the other six isoforms during epithelial cell invasion will be informative for our understanding of how *P. aeruginosa* usurps this host cofactor to promote virulence.

Previous investigations exploring how exotoxins regulate *P. aeruginosa* epithelial cell invasion attributed ExoS and ExoT GAP and ADPRT domain functions to controlling the pathogen’s ability to invade or remain extracellular. From these studies, the general conclusions were that GAP domain function prevented invasion through host GTPase-mediated cytoskeletal destabilization (12, 13, 18, 19), while the ADPRT domain ADP-ribosylated numerous host cell targets impacting various processes and, incidentally, was also found to support intracellular survival and replication (should it occur) (10, 32, 33, 47). However, experiments supporting this idea counted intracellular CFU through gentamicin protection assays, which would fail to distinguish between many invasion events versus rapid replication post-invasion. Here, we expand on these studies with novel findings that clarify how ExoS and ExoT contribute to intracellular populations of *P. aeruginosa*. By using a fluorescent dual-reporter plasmid that allowed us to visualize all internalized bacteria through constitutive GFP expression, as well as monitor T3SS activation through P_exoS_-driven mScarlet expression, we found that individual ExoS/T GAP or ADPRT domains do not significantly alter the frequency of invaded cells relative to WT PAO1 (Fig. 4D). Rather, we observed differences in the relative frequencies of *P. aeruginosa* confined to vacuoles (Fig. 4E) or replicating in the cytoplasm (Fig. 4F). Strains that expressed only the ExoS or ExoT GAP domain were both capable of invasion but were primarily retained in vacuoles with no T3SS transcriptional activity (Fig. 4E and F). The use of the fluorescent dual-reporter plasmid here allowed us to observe and track the population of T3SS⁻ *P. aeruginosa* that were previously untraceable in studies using T3SS-inducible GFP reporter plasmids (47). Stemming from these advances, we found that expression of the ExoS ADPRT domain could autonomously drive vacuolar exit, T3SS transcriptional activation, and cytoplasmic replication (Fig. 4E and F). Conversely, we found that expression of the ExoT ADPRT domain led to less vacuolar escape and replication compared to ExoS ADPRT, more closely matching the vacuole escape frequency observed in WT PAO1 (Fig. 4E and F), although this difference was not apparent in primary cells (Fig. 8). Finally, by performing infections with strains in which a single exotoxin domain is catalytically inactivated, we found that loss of only the ExoS ADPRT domain led to the most drastic shift toward *P. aeruginosa* remaining confined to an intracellular vacuole in a T3SS⁻ state (Fig. 5).

The exception to these data, which remains enigmatic, was the enhanced intracellular replication of the Δ*exoSTY* mutant (Fig. 4A) (9, 47). If ExoS ADPRT is needed to promote vacuole exit, how would an effector-null strain exit vacuoles? Here, we consider the idea of heterogeneous T3SS expression during host cell contact. First, T3SS^+^ bacteria do not appear to enter cells; i.e., if an individual bacterium is actively secreting effectors, it is not taken up (Fig. 9A) (47). This is where the ∆*exoSTY* mutant is unique: it can be internalized while in a T3SS^+^ state, as no effectors opposing this activity are produced (Fig. 4A and 9A). Second, by diminishing the probability of T3SS activation through our high MOI experiments (Fig. 6), we find that even the ∆*exoSTY* mutant remains confined in vacuoles and T3SS⁻. This suggests that once a T3SS⁻ bacterium is retained in a vacuole, it appears to be trapped in that state unless acted upon by ExoS ADPRT introduced into the cytoplasm of the same cell. Thus, rapid exit of the ∆*exoSTY* mutant may be attributed to the fact that it can be internalized while in a T3SS^+^ state. Our data support the idea that expression of the T3SS from within a vacuole leads to escape (compare Fig. 7C and G).

A recent publication by Schator et al. demonstrated that vacuolar exit of intracellular *P. aeruginosa* can be modulated by the extracellular subpopulation in both T3SS effector-independent and -dependent pathways (50). Using a Δ*exsE* mutant that constitutively expresses the T3SS apparatus and effectors, they show intracellular vacuolar escape is mediated on two fronts: a Ca^2+^-channel–independent mechanism in which pores formed by the T3SS needle in corneal epithelial cell membranes cause an influx of Ca^2+^ and vacuolar destabilization, and a Ca^2+^-channel–dependent mechanism influenced by the presence of the T3SS exotoxins (61). Coincidentally, we arrived at a similar observation of cooperation between extracellular *P. aeruginosa* and intracellular vacuolar escape in HeLa cells, but provide additional mechanistic insight into roles of T3SS exotoxins and the specific importance of the ExoS ADPRT domain, as well as a method to visualize the T3SS state of intracellular populations. As a tool to probe intracellular populations of *P. aeruginosa*, we report and substantiate the use of a high MOI to circumvent extracellular T3SS activation (Fig. 6). By increasing the inoculum of infection to an MOI of 1,000, we effectively inhibit T3SS activation (Fig. 6B, C and F) while also increasing the frequency of invasion, with nearly all cells containing multiple vacuoles harboring T3SS⁻ *P. aeruginosa* (Fig. 6B, D and E). We hypothesize that T3SS inhibition is mediated through the Rhl quorum sensing system, but that remains to be tested (62, 63). Then, we found that extracellular delivery of ExoS ADPRT promotes intracellular vacuolar escape and cytoplasmic replication (Fig. 7C and D). However, it appears that the ability to express the T3SS apparatus is also important for optimal exit from the vacuole, as we observed a much lower frequency of escape for an Δ*exsA* mutant upon secondary infection with an ExoS ADPRT-expressing strain (Fig. 7G and H). The underlying mechanism of this T3SS activation within the vacuolar population upon extracellular ExoS ADPRT delivery is not yet understood, but it is plausible that ADP-ribosylation of specific host target proteins or potentially Ca^2+^-channel activity could be important in triggering vacuolar T3SS activation. With regard to the T3SS apparatus pore-driven vacuolar escape mechanism shown by Schator et al., we found no discernible influence of secondary infection with an Δ*exoSTY* mutant on vacuolar exit (Fig. 7C, D, G and H). These differences could be attributed to our use of a Δ*exoSTY* mutant (non-constitutive T3SS expression) rather than a Δ*exsE* (constitutive T3SS expression) or differences between cell types.

Taken together, our study furthers our understanding of the molecular mechanisms controlling *P. aeruginosa* intracellular localization and the T3SS exotoxin’s contributions in determining vacuolar residence and cytoplasmic replication. Our data provide foundational evidence for these newly appreciated roles of ExoS and ExoT and have broad implications for our understanding of *P. aeruginosa* intracellular infection dynamics. Additionally, this study further highlights the existence of a long-lasting, T3SS⁻ vacuolar subpopulation of *P. aeruginosa* that warrants the interrogation of these bacteria as persisters.

## MATERIALS AND METHODS

### Bacterial strains and cell lines

Wild-type *P. aeruginosa* PAO1 was used to generate isogenic mutants of Δ*exoSTY*, Δ*exoSY*, and Δ*exoTY* (64), as well as all point mutant strains for ExoS GAP⁻ (R146K), ExoS ADPRT⁻ (E379D, E381D), ExoT GAP⁻ (R149K), and ExoT ADPRT⁻ (E383D, E385D) in an *exoY* (K81M) mutant background. The T3SS dual-reporter plasmid, pCG-P_exoS_-mS, which confers constitutive GFP expression and *exoS* promoter-driven expression of mScarlet, was used for visualization during infection (44). All strains were grown on tryptic soy agar plates at 37°C unless indicated otherwise. Plasmid selection was maintained using 100 μg/mL gentamicin. HeLa cells were cultured in phenol red-free Dulbecco’s Modified Eagle Medium (DMEM; Gibco) supplemented with 10% fetal bovine serum (FBS) at 37°C 5% CO_2_. Primary corneal epithelial cells (normal, human, ATCC PCS-700-010) were cultured in ATCC Corneal Cell Basal Medium containing supplements from the Corneal Epithelial Cell Growth Kit. Passaging was performed using protocols from the distributor.

*Escherichia coli* DH5α was used for cloning, and *lysY/I*^q^ was used for expression and purification of both WT and null versions of ExoS and ExoT, as well as human 14-3-3 isoform β. *E. coli* cultures were grown in Luria broth (LB) or LB agar plates supplemented with ampicillin (100 μg/mL), when indicated. Bacterial strains and plasmids used in this study are listed in Table 2.

**TABLE 2:** Bacterial strains and plasmids used in this study

Strains	Description	Reference or source
*E. coli* DH5α	Cloning strain	New England Biolabs
*E. coli* SM10	Conjugation strain	John Mekalanos (Harvard Medical School)
*E. coli lysY/I* ^q^	Protein expression and purification strain	New England Biolabs
*P. aeruginosa* PAO1 (PAO1F)	Wildtype *P. aeruginosa*	Arne Rietsch (Case Western Reserve University) (62)
Δ*exoSTY*	*exoSTY* mutant	Arne Rietsch (Case Western Reserve University) (65)
Δ*exoSY*	*exoSY* mutant	Arne Rietsch (Case Western Reserve University) (65)
Δ*exoTY*	*exoTY* mutant	Arne Rietsch (Case Western Reserve University) (65)
*exoY⁻*	*exoY* (K81M) mutant	This study
*exoS* GAP⁻ *exoY⁻*	*exoS* (R146K) *exoY* (K81M) mutant	This study
*exoS* ADPRT⁻ *exoY⁻*	*exoS* (E379D, E381D) *exoY* (K81M) mutant	This study
*exoS* GAP⁻/ADPRT⁻ *exoY⁻*	*exoS* (R146K, E379D, E381D) *exoY* (K81M) mutant	This study
*exoT* GAP⁻ *exoY⁻*	*exoT* (R149K) *exoY* (K81M) mutant	This study
*exoT* ADPRT⁻ *exoY⁻*	*exoT* (E383D, E385D) *exoY* (K81M) mutant	This study
*exoT* GAP⁻/ADPRT⁻ *exoY⁻*	*exoT* (R149K, E383D, E385D) *exoY* (K81M) mutant	This study
*exoS* GAP⁻ *exoT* ADPRT⁻ *exoY⁻*	*exoS* (R146K) *exoT* (E383D, E385D) *exoY* (K81M) mutant	This study
*exoS* ADPRT⁻ *exoT* GAP⁻ *exoY⁻*	*exoS* (E379D, E381D) *exoT* (R149K) *exoY* (K81M) mutant	This study
*exoS* GAP⁻ *exoT* GAP⁻/ADPRT⁻ *exoY⁻*	*exoS* (R146K) *exoT* (R149K, E383D, E385D) *exoY* (K81M) mutant	This study
*exoS* ADPRT⁻ *exoT* GAP⁻/ADPRT⁻ *exoY⁻*	*exoS* (E379D, E381D) *exoT* (R149K, E383D, E385D) *exoY* (K81M) mutant	This study
*exoS* GAP⁻/ADPRT⁻*exoT* GAP^-^ *exoY^-^*	*exoS* (R146K, E379D, E381D) *exoT* (R149K) *exoY* (K81M) mutant	This study
*exoS* GAP⁻/ADPRT⁻*exoT* ADPRT⁻ *exoY⁻*	*exoS* (R146K, E379D, E381D) *exoT* (E383D, E385D) *exoY* (K81M) mutant	This study

Plasmids	Description	Reference or source
pET15b	N-terminal 6×His-tag protein expression vector	Novagen (Sigma-Aldrich; 69,661)
*exoS_70-453_*-HA-pET15b	*exoS_70-453_*-HA cloned into NdeI and BamHI sites of pET15b	This study
*exoS_70-453_*-GAP⁻- HA-pET15b	*exoS_70-453_*-HA with GAP^-^ (R146K) point mutation cloned into NdeI and BamHI sites of pET15b	This study
*exoT_70-457_*-FLAG-pET15b	*exoT_70-457_*-FLAG cloned into NdeI and BamHI sites of pET15b	This study
*exoT_70-457_*-ADPRT⁻-FLAG-pET15b	*exoT_70-457_*-FLAG with ADPRT^-^ (E383D, E385D) point mutations cloned into NdeI and BamHI sites of pET15b	This study
pCG-P_exoS_-mS	Dual-reporter plasmid with constitutive *lacUV5*-driven GFP expression and P*exoS* inducible expression of mScarlet	Christopher Corcoran (Loyola University Chicago) (44)
pEXG2	Integrating suicide plasmid	Arne Rietsch (Case Western Reserve University) (66)
pEXG2-*exoS* GAP⁻	*exoS* containing GAP (R146K) point mutation cloned into pEXG2	This study
pEXG2-*exoS* ADPRT⁻	*exoS* containing ADPRT (E379D, E381D) point mutations cloned into pEXG2	This study
pEXG2-*exoS* GAP⁻/ADPRT⁻	*exoS* containing GAP (R146K) and ADPRT (E379D, E381D) point mutations cloned into pEXG2	This study
pEXG2-*exoT* GAP⁻/ADPRT⁻	*exoT* containing GAP (R149K) and ADPRT (E383D, E385D) point mutations cloned into pEXG2	This study
YWHAB	14-3-3 β with C-terminal 6×His-tag cloned into pTvHR21-SGC	Addgene (#39128)

### Generation of ExoS and ExoT point mutants

Point mutants in the GAP and ADPRT domains of ExoS and ExoT were generated by allelic exchange using mutant constructs cloned into an integrating suicide plasmid, pEXG2. For *exoT*, constructs containing point mutations in the GAP⁻ (R149K) and ADPRT⁻ (E383D, E385D) domains were prepared using a Q5 Site-Directed Mutagenesis Kit (NEB). This DNA construct was amplified using ExoT_HindIII_F and ExoT_BamHI_R primers (Table 3), ligated into the HindIII and BamHI sites of pEXG2, and transformed into *E. coli* DH5α. For *exoS*, point mutant constructs for *exoS* GAP⁻ (R146K), ADPRT⁻ (E379D, E381D), and GAP⁻/ADPRT⁻ were amplified using exoS_pEXG2_F and exoS_pEXG2_R primers and ligated into KpnI and BamHI sites of pEXG2. Plasmids bearing *exoS* and *exoT* point mutation constructs were verified by DNA sequencing.

**TABLE 3:** Primers used in this study

Name	Sequence (5′ → 3′)
exoS70_F_NdeI	CAGTCACATATGGACTGGCTGGGCAAGCTGTTGG
exoS_HA_R_BamHI	CTGACTGGATCCTTAAGCGTAATCTGGAACATCGTATGGGTATCCGCTGCTGGCCAGATCAAGGCCGCGCATC
exoT70_F_NdeI	CATGCATATGGAGTGGCTGGGCAAACTGCTGGGG
exoT_FLAG_R_BamHI	CATGGGATCCTTACTTGTCGTCATCGTCTTTGTAGTCGGCCAGGTCGAGGCCGCG
exoT_HindIII_F	CATGAAGCTTGCGCCTCGTTCCTAGACTGG
exoT_BamHI_R	CATGGGATCCCGGCCTTTGGAAATCCGAAACAA
exoS_F	TATACAGGAGAAGGCAACCATCATG
exoS_R	TATAACGTCTTTCTTTTACGACCGG
exoT_E383D_E385D_F	TCGATCGAGGGCGATGATCAGTAGATCCTCTACGAC
exoT_E383D_E385D_R	GTCGTAGAGGATCTACTGATCATCGCCCTCGATCGA
exoT_R149K_F	GCGACGGCGCTCTGAAGTCGCTGGCCACCGC
exoT_R149K_R	GCGGTGGCCAGCGACTTCAGAGCGCCGTCGC
exoS_pEXG2_F	TATAGGTACCATGCATATTCAATCGCTT
exoS_pEXG2_R	ATATGGATCCTCAGGCCAGATCAAGGCC

pEXG2 plasmids with *exoT* and *exoS* point mutations were transformed into *E. coli* SM10, which were subsequently used to transfer the plasmids into *P. aeruginosa* PAO1 by conjugation. Briefly, conjugations were performed on LB agar, followed by counter-selection on yeast extract and tryptone (YT) agar plates supplemented with 15% sucrose. Point mutation candidates were verified using PCR amplicon sequencing.

Parental PAO1 WT or specific mutant genomic DNA samples, which had undergone the maximum number of sequential allelic exchange mutagenesis events to nullify four T3SS exotoxin catalytic activities, were submitted to Plasmidsaurus (San Francisco, CA) for whole-genome sequencing using Oxford Nanopore Technology. Reads were aligned to the PAO1 reference genome (GCF_000006765.1) and analyzed for sequence variations using breseq (v0.39.0) (67). FastQ files were submitted to the NCBI Sequence Read Archive and can be accessed at the BioProject database under ID PRJNA1423707: http://www.ncbi.nlm.nih.gov/bioproject/1423707.

### Host cell infections

One day prior to infection, HeLa cells were seeded into an uncoated Ibidi μ-Slide 8 Well High IbiTreat at 1.0 × 10^5^ cells/well in 500 μL DMEM supplemented with 10% FBS. The next day, *P. aeruginosa* strains were prepared by resuspending a loopful of culture in 1 mL PBS and diluted 1:10 to measure OD_540_. Thirty minutes prior to infection, the medium was changed to DMEM containing 10% FBS and Hoechst (0.6 μg/mL). Cells were then infected at an MOI of 10 for 3 h and incubated at 37°C 5% CO_2_. The medium was then changed to DMEM containing 10% FBS, amikacin (0.2 mg/mL), and polymyxin B (10 μg/mL) and incubated for 1 h, and cells were imaged every 30 min for 10 h. For sequential infections, cells were first infected at an MOI of 1,000 for 2 h. A medium change to eliminate extracellular bacteria was performed as described above. After 1 h of antibiotic treatment, cells were washed twice with 1× PBS, DMEM containing 10% FBS was replaced, and cells were secondarily infected with indicated strains at an MOI of 10 for 3 h. The medium was changed to include antibiotics, and cells were imaged every 30 min for 14 h.

Experiments to measure HeLa cell death rates were performed identically with non-fluorescent bacteria, but included both Hoechst and propidium iodide to detect nuclei of dead (permeable) cells. Death rates were quantified as previously described (9), using a script available in the following GitHub repository: https://github.com/Llamero/Nuclei_analysis-macro.

Primary corneal epithelial cells were seeded at 6 × 10^4^ cells/well in 500 μL ATCC Corneal Cell Basal Medium containing supplements from the Corneal Epithelial Cell Growth Kit. The experiments were conducted identically to those in HeLa cells using the appropriate media for the cell type and excluding the use of Hoechst.

### Microscopy and image analysis

Images were captured on a Nikon Ti-E inverted microscope equipped with an X-Cite XYLIS XT720S Broad Spectrum LED Illumination System using Nikon Perfect Focus, an Okolab stage-top incubation chamber at 37°C with 5% CO_2_, a DS-Qi2 CMOS camera, and a CFI Plan Apochromat Lambda D 40× air NA 0.95 objective (for HeLa cells). In some replicates, corneal epithelial cells were imaged using a CFI Plan Apochromat Lambda D 20× NA 0.8 air objective to accommodate more cells per field of view, due to heterogeneity in cell size. Fields for time-lapse imaging were selected between 3 and 4 h post-infection without viewing fluorescence channels to limit bias in field selection. Eight fields per condition were imaged every 30 min, beginning at 4 h post-infection and ending at either 14 or 18 h post-infection.

Time-lapse images were scored manually. A cell was defined as “invaded” if, throughout the time series, it contained stable fluorescent bacteria within its boundaries, as viewed by differential interference contrast imaging. If the bacteria remained in a GFP + puncta, that was scored as a cell containing “GFP + vacuoles.” If the cell contained mScarlet + bacteria, that was scored as exhibiting “T3SS transcriptional activity.” Some cells contained both populations. The number of cells containing bacteria was reported as a percent of total cells in the field. SuperPlots to illustrate variation among fields and between biological replicates were prepared in GraphPad Prism using the guide provided by reference 68.

### Immunofluorescent staining for ADP-ribosylation

Collagen-coated coverslips in 24-well plates were seeded with 1.5 × 10^5^ HeLa cells and allowed to adhere for at least 16 h. The next day, cells were infected at an MOI of 10 or 1,000 for 3 h. Following infection, the medium was changed to DMEM + 10% FBS + amikacin (0.2 mg/mL) + polymyxin B (10 μg/mL) and incubated for 1 h. Cells were washed twice with PBS and then permeabilized with 4% paraformaldehyde for 10 min at room temperature. Cells were washed twice with PBS, quenched with 150 mM glycine for 10 min at room temperature, and then washed twice with PBS. Cells were incubated in blocking solution (5% FBS, 2.5% gelatin, 0.1% Triton X-100, 0.05% Tween-20 in PBS) for 1 h at room temperature, protected from light. Blocking solution was replaced with antibody solution (2.5% FBS, 1.25% gelatin, 0.1% Triton X-100, 0.05% Tween-20 in PBS) containing 1:1,000 α-mono-ADP-ribose binding reagent (Millipore Sigma; MABE1076) and incubated overnight at 4°C, protected from light. Cells were washed four times with 1× PBS and then incubated with 1:1,000 AlexaFluor 647 goat-α-rabbit IgG (H + L) (Invitrogen; A21245) for 1 h at room temperature. After a single 5-minute PBS wash, cells were incubated with a DAPI solution in PBS (0.2 μg/mL) for 5 min, followed by two additional 5-minute PBS washes. Coverslips were mounted on slides with ProLong Diamond Antifade Mountant (Invitrogen; P36965) and allowed to cure overnight at room temperature prior to imaging.

### Protein purification

The expression vectors used to purify His_6_-ExoS_70-453_-HA and His_6_-ExoT_70-457_-FLAG were constructed in pET15b as follows. *exoS* and *exoT* were amplified from PAO1 gDNA via PCR using primer pairs exoS70_F_NdeI/exoS_HA_R_BamHI and exoT70_F_NdeI/exoT_FLAG_R_BamHI, respectively, which exclude the first 70 codons encoding an unstructured membrane-localization domain (16). The same primer sets were used to amplify *exoS*70-GAP⁻ and *exoT*70-ADPRT⁻ from the corresponding PAO1 mutant gDNA. These constructs were inserted into the NdeI and BamHI sites of pET15b and verified via DNA sequencing.

*E. coli* lysY/I^q^ strains harboring *exoS_70-453_*-HA-pET15b, *exoT_70-457_*-FLAG-pET15b, *exoS_70-453_*-GAP⁻-HA-pET15b, *exoT_70-457_*-ADPRT⁻-FLAG-pET15b, or YWHAB (Addgene; #39128) were cultured in 1 L LB supplemented with ampicillin (100 μg/mL) at 37°C with shaking at 200 rpm until an OD_600_ of 0.2–0.3 was achieved, and protein expression was induced by adding 0.5 mM isopropyl-β-D-thiogalactopyranoside (IPTG; GoldBio) for 3 h. Cultures were pelleted by centrifugation at 8,000 rpm for 10 min at 4°C. Pellets were resuspended in 50 mL of 50 mM Tris-HCl, 300 mM NaCl, pH 8.0, containing 25 mM imidazole (MP Bio), 1 mM phenylmethylsulfonyl fluoride (PMSF; Thermo Fisher), and 1 cOmplete Mini EDTA-free protease inhibitor tablet (Sigma). Cells were lysed using a Branson Sonifier 450 at a constant rate of 10 s per pulse at 80% amplitude in 2-minute intervals for a total of 10 min on ice. Lysates were clarified by centrifugation at 12,000 rpm at 4°C, and subsequently filtered through a 0.45 μm pore syringe filter (Fisher). Filtered lysates were incubated with 1 mL of pre-equilibrated nickel-nitriloacetic acid (NTA) resin slurry (Qiagen) in 50 mM Tris-HCl, 300 mM NaCl, pH 8.0, with 25 mM imidazole for 1 h at 4°C on a rotisserie. Lysate resin slurry was poured into a glass chromatography column, and the resin bed was washed with 10 column volumes of 50 mM Tris-HCl, 300 mM NaCl, pH 8.0, with 25 mM imidazole. Proteins were eluted with 5 mL 50 mM Tris-HCl, 300 mM NaCl, pH 8.0, with 500 mM imidazole, and dialyzed using 10 kDa MW cutoff regenerated cellulose dialysis tubing (Spectrum). Dialysis was performed in phases to taper off imidazole concentrations of 50 mM Tris-HCl, 300 mM NaCl, pH 8.0 buffer at 4°C as follows: 100 mM imidazole for 4 h, 25 mM imidazole for 16 h, and 0 mM imidazole for 4 h, repeated twice.

Dialyzed proteins were concentrated to ~5 mg/mL using Amicon Ultra-15 centrifugal filters (Sigma) and clarified by centrifugation at 14,000 rpm at 4°C for 10 min to perform size-exclusion chromatography using a Cytiva Äkta Pure 25 chromatography system. Samples (500 μL volume) were applied to a Superose 6 Increase 10/300 GL column (Cytiva) equilibrated in 50 mM Tris-HCl pH 8.0, 300 mM NaCl, and separated at a flow rate of 0.4 mL/min. Fractions of 0.5 mL were collected. Purity of protein fractions was analyzed by SDS-PAGE and GelCode Blue protein staining (Thermo Fisher), and concentration was determined using Qubit (Thermo Fisher). Aliquots were stored at −80°C until use.

### *In vitro* ADP-ribosylation assays

Two micrograms of purified ExoS, ExoT, or both were mixed with 2 μg of 14-3-3 β, with or without the addition of 1 mM NAD^+^, and brought up to 15 μL in 10 mM Tris, 20 mM NaCl, pH 8.0. Reactions were incubated at room temperature or 37°C for 1 h, unless indicated otherwise. Reactions were then boiled for 10 min at 100°C with 4× Laemmli sample buffer (Bio-Rad) containing β-mercaptoethanol. To visualize band shifts indicative of protein modification, 5 μg of ExoS, ExoT, or both were used in reactions under the same conditions as above and analyzed by SDS-PAGE followed by GelCode Blue protein staining.

### RhoA GTPase activity assays

*In vitro* ADP-ribosylation assays were performed as described above using 1 μg of purified ExoS, ExoT, or both. Reactions were incubated at room temperature for 2 h. GAP activity toward recombinant human RhoA (Abcam; AB268932) was measured using a Promega GTPase-Glo Assay Kit following the manufacturer’s protocol. Briefly, 100 ng of proteins from *in vitro* ADP-ribosylation assays were incubated with 100 ng of RhoA in GTPase buffer with 5 μM GTP and 1 mM DTT for 1 h at 37°C. GTPase-Glo reagent was added, and reactions were incubated for 30 min at room temperature with shaking at 250 rpm. Detection reagent was added to reactions, and luminescence was measured using a BioTek Synergy H1 microplate reader.

### Western blots

*In vitro* ADP-ribosylation reactions were resolved in a 12% polyacrylamide gel and transferred to 0.2 μm pore polyvinylidene difluoride (PVDF) membrane in Tris-glycine buffer using a Bio-Rad Trans-Blot Turbo system. Membranes were blocked for 1 h in 5% bovine serum albumin (BSA) in Tris-buffered saline + 0.1% Tween 20 (TBST). For all blots, membranes were incubated with primary antibody overnight at 4°C, washed three times with TBST, incubated with secondary antibody for 1 h at room temperature, and then washed three times with TBST again before development. For detection of mono-ADP-ribosylation, membranes were incubated with 1:6,000 α-mono-ADP-ribose binding reagent (Millipore Sigma; MABE1076) and then 1:3,000 goat-α-rabbit IgG (H + L) HRP conjugate (Bio-Rad; #1706515). For detection of HA-tagged ExoS, membranes were incubated with 1:5,000 rat α-HA (Millipore Sigma; #11867423001), followed by 1:3,000 goat-α-rat IgG HRP conjugated (R&D; HAF005). For detection of FLAG-tagged ExoT, membranes were incubated with 1:10,000 mouse α-FLAG (Millipore Sigma; F3165), followed by goat-α-mouse IgG (H + L) HRP conjugate (Bio-Rad; #1706516). Blots were developed using Bio-Rad Clarity Western ECL Substrate and imaged on a Bio-Rad ChemiDoc XRS+.

To validate the secretion of catalytic null exotoxins, *P. aeruginosa* strains were grown overnight in 5 mL TSB and subcultured 1:100 in 5 mL TSB supplemented with 100 mM monosodium glutamate and 1% glycerol, plus 2 mM EGTA. Subcultures were grown at 37°C, shaking at 220 rpm for 6 h, and OD_540_ was recorded for each strain. Cultures were then centrifuged at 4,000 rpm for 10 min at 4°C, and 1.35 mL of clarified supernatant was transferred to 2 mL tubes, followed by the addition of 150 μL of 100% TCA. Tubes were mixed by inverting and incubated overnight at 4°C. Precipitated proteins were pelleted by centrifugation at 13,000 rpm for 15 min at 4°C and washed twice with 100% ethanol. Following the washes, pellets were dried for 30 min at room temperature and resuspended in 30 μL of a 1:1 mixture of 0.5 M Tris-HCl pH 8.0 + 4% SDS and 2× Laemmli buffer + β-mercaptoethanol (Bio-Rad). Samples were boiled for 10 min at 100°C, and loading volumes were normalized to the highest culture OD_540_. Western blots were performed as described above. For detection of ExoS and ExoT, membranes were incubated with 1:4,000 rabbit α-ExoT antisera, which cross-reacts with ExoS due to high similarity (36), followed by 1:5,000 goat-α-rabbit IgG (H + L) HRP conjugate (Bio-Rad; #1706515). Blots were developed using Bio-Rad Clarity Western ECL Substrate and imaged on a Bio-Rad ChemiDoc XRS+.

### Liquid chromatography-tandem mass spectrometry

Proteins from *in vitro* ADP-ribosylation assays were subjected to reduction with 5 mM dithiothreitol (DTT) for 45 min at room temperature and alkylation with 15 mM iodoacetamide for 20 min at room temperature, protected from light. DTT concentration was then adjusted to 10 mM to remove excess iodoacetamide for 20 min. Peptides were digested with 300 ng trypsin (Promega) and incubated at 37°C, shaking at 200 rpm for 18 h. Digested peptides were dried in a SpeedVac vacuum concentrator and resuspended in 10 μL of 0.15% trifluoroacetic acid (TFA). Peptides were purified using Pierce C18 10 μL tips (Thermo Fisher) following the manufacturer’s protocol and eluted in 10 μL 0.1% TFA in 70% acetonitrile, then dried.

Purified, dried peptides were resolubilized in 5% acetonitrile with 0.1% formic acid solution, and half of the sample was loaded onto a Vanquish Neo UHPLC system (Thermo Fisher) using a heated trap-and-elute workflow with a C18 PepMap, 0.3 mm × 5 mm, 5 µM trap column (Thermo Fisher; 160454) in a forward-flush configuration, connected to a 50 cm EASY-Spray analytical column (Thermo Fisher; ES75500PN) 2 µM, 100 Å, 75 µm × 500 mm, with 100% Buffer A (0.1% formic acid in water), a flow rate of 0.300 µL, and the column oven operating at 35°C. Peptides were eluted over a 60-minute gradient using 80% acetonitrile with 0.1% formic acid (buffer B), increasing from 5% to 35% over 36 min, to 50% over 10 min, then to 99% over 2 min, and held at 99% for 7 min, after which all peptides were eluted. Spectra were acquired on an Orbitrap Eclipse.

A Tribrid mass spectrometer (Eclipse) with FAIMS Pro interface (Thermo Fisher) running Tune 3.5 and Xcalibur 4.5 was used for all acquisition methods, with spray voltage set to 1,800 V and ion transfer tube temperature set at 300°C. FAIMS was switched between CVs of −45 V, –55 V, and −65 V with cycle times of 1 s. MS1 spectra were acquired at 120,000 resolutions with a scan range of 375–1,500 m/z, normalized AGC target of 300%, and maximum injection time set to auto; S-lens RF level set to 30, without source fragmentation; and data type set to positive, profile. Precursors were filtered using monoisotopic peak determination set to peptide MIPS, including charge states 2–7 and rejecting unassigned precursors, while dynamic exclusion was enabled with n = 1 for a 60-second exclusion duration at 10 ppm tolerance for both high and low mass ranges. For the DD-MS2 scan, the isolation mode was set to quadrupole with a 1.6 m/z isolation window, the activation type was HCD with 30% collision energy, the detector type was ion trap with rapid scan rate, AGC target was set to 100%, maximum injection time was 35 ms, microscans were set to 1, and the data type was centroid.

### LC-MS/MS data analysis

Raw data were analyzed using Proteome Discoverer 2.5 (Thermo Fisher) with the Sequest HT search engines. The data were searched against the entries in the UniProt protein sequence P31946, human 14-3-3 protein beta/alpha (YWHAB), and *Pseudomonas aeruginosa* proteins Q9I78, Exoenzyme T (*exoT*), and Q51451 Exoenzyme S (*exoS*), with the *E. coli* Proteome database as background. The Sequest search parameters included precursor mass tolerance of 10 ppm and 0.6 Da for fragments, up to four missed trypsin cleavages, oxidation (Met), acetylation (protein N-term), and ADP-ribosylation (Arg) as variable modifications, and carbamidomethylation (Cys) as a static modification. Percolator PSM validation was used with the following parameters: strict false discovery rate (FDR) of 0.01, relaxed FDR of 0.05, maximum ΔCn of 0.05, and validation based on *q*-value. Precursor ion quantifier settings were as follows: Peptides to Use: Unique + Razor; Consider Protein Groups for Peptide Uniqueness set as True; Precursor Abundance Based On: Intensity. This data is available at ftp://massive-ftp.ucsd.edu/v10/MSV000098404/.

## SUPPLEMENTAL MATERIAL

Supplemental material iai.00267-26-s0001.docx Fig. S1 to S3; Table S1; legends for Supplemental movies.

Movie S1 iai.00267-26-s0002.mp4 HeLa cell infection with *P. aeruginosa* T3SS mutants. HeLa cells were infected with WT PAO1, a strain lacking exotoxins S, T, and Y (ΔSTY), or a T3SS-deficient mutant (Δ*exsA*) at an MOI of 10 for 3 hours. At 3 hpi, amikacin (0.2 mg/mL) and polymyxin B (10 μg/mL) were supplemented and cells were imaged every 30 minutes for 10 hours.

Movie S2 iai.00267-26-s0003.mp4 HeLa cell infection with *P. aeruginosa* expressing only ExoS functional domains. HeLa cells were infected with a *P. aeruginosa* strain expressing only ExoS (ΔTY), or versions of ExoS where only the GAP or ADPRT domains are catalytically active at an MOI of 10 for 3 hours. At 3 hpi, amikacin (0.2 mg/mL) and polymyxin B (10 μg/mL) were supplemented and cells were imaged every 30 minutes for 10 hours.

Movie S3 iai.00267-26-s0004.mp4 HeLa cell infection with *P. aeruginosa* expressing only ExoT functional domains. HeLa cells were infected with a *P. aeruginosa* strain expressing only ExoT (ΔSY), or versions of ExoT where only the GAP or ADPRT domains are catalytically active at an MOI of 10 for 3 hours. At 3 hpi, amikacin (0.2 mg/mL) and polymyxin B (10 μg/mL) were supplemented and cells were imaged every 30 minutes for 10 hours.

Movie S4 iai.00267-26-s0005.mp4 HeLa cell infection with *P. aeruginosa* ExoS catalytic null mutants. HeLa cells were infected with a *P. aeruginosa* strain where only the GAP and/or ADPRT domains of ExoS are catalytically inactivated at an MOI of 10 for 3 hours. At 3 hpi, amikacin (0.2 mg/mL) and polymyxin B (10 μg/mL) were supplemented and cells were imaged every 30 minutes for 10 hours.

Movie S5 iai.00267-26-s0006.mp4 HeLa cell infection with *P. aeruginosa* ExoT catalytic null mutants. HeLa cells were infected with a *P. aeruginosa* strain where only the GAP and/or ADPRT domains of ExoT are catalytically inactivated at an MOI of 10 for 3 hours. At 3 hpi, amikacin (0.2 mg/mL) and polymyxin B (10 μg/mL) were supplemented and cells were imaged every 30 minutes for 10 hours.

Movie S6 iai.00267-26-s0007.mp4 HeLa cell infection with *P. aeruginosa* ExoS and ExoT combination catalytic null mutants. HeLa cells were infected with a *P. aeruginosa* strain where a single functional domain of both ExoS and ExoT are catalytically inactivated at an MOI of 10 for 3 hours. At 3 hpi, amikacin (0.2 mg/mL) and polymyxin B (10 μg/mL) were supplemented and cells were imaged every 30 minutes for 10 hours.

Movie S7 iai.00267-26-s0008.mp4 Increasing the MOI represses *P. aeruginosa* T3SS activation. HeLa cells were infected with WT PAO1 at MOI 10, 100, or 1000 for 3 hours. At 3 hpi, cells were treated with amikacin (0.2 mg/mL) and polymyxin B (10 μg/mL) and imaged every 30 minutes for 14 hours.

Movie S8 iai.00267-26-s0009.mp4 Extracellular T3SS delivery of ExoS ADPRT promotes intracellular vacuolar escape. HeLa cells were infected with an ExoS GAP^+^-expressing strain (upper panels), or with a ∆*exsA* mutant (lower panels), each transformed with pCG-P_exoS_-mS, at an MOI 1000 for 2 hours. At 2 hpi, cells were treated with amikacin (0.2 mg/mL) and polymyxin B (10 μg/mL) for 1 hour, and then subsequently infected with indicated non-fluorescent mutant strain expressing a single active ExoS or ExoT domain at a MOI of 10 for 3 hours. Infected cells were treated again with antibiotics to eliminate extracellular bacteria and imaged every 30 minutes for 14 hours.

Movie S9 iai.00267-26-s0010.mp4 Primary human corneal epithelial cell infection with *P. aeruginosa* T3SS mutants. Corneal cells were infected with WT PAO1, a strain lacking exotoxins S, T, and Y (ΔSTY), or a T3SS-deficient mutant (Δ*exsA*) at an MOI of 10 for 3 hours. In the lower panels, corneal cells were infected with strains where only indicated single catalytic activities from either ExoS or ExoT were present. At 3 hpi, amikacin (0.2 mg/mL) and polymyxin B (10 μg/mL) were supplemented and cells were imaged every 30 minutes for 10 hours.

ASM does not own the copyrights to Supplemental Material that may be linked to, or accessed through, an article. The authors have granted ASM a non-exclusive, world-wide license to publish the Supplemental Material files. Please contact the corresponding author directly for reuse.
