# Supplementary material for: Individual contributions of exotoxins S and T on internalized P. aeruginosa
Source: Infect Immun. 2026 Jun 15;94(7):e00267-26. doi: 10.1128/iai.00267-26 (PMC13367040; doi:10.1128/iai.00267-26)
Supplement: Supplemental material — Fig. S1 to S3; Table S1; legends for Supplemental movies. [file iai.00267-26-s0001.docx]

**Supplemental Figure 1**


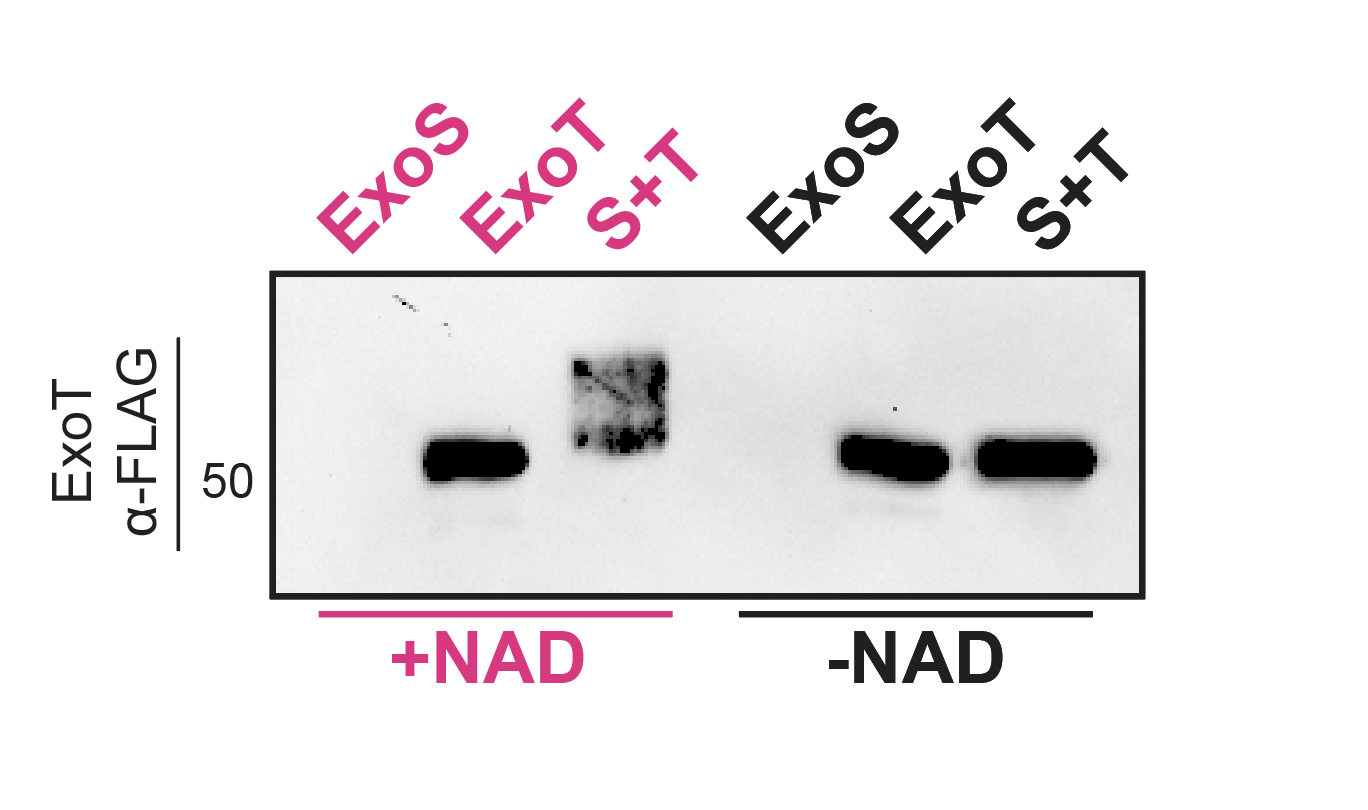


**Figure S1. ExoT is cross-ADP-ribosylated by ExoS at 37°C.** *In vitro* ADP-ribosylation assays were performed using 2 μg of each HA-ExoS and/or FLAG-ExoT and 14-3-3 β with or without the addition of NAD^+^ and incubated for 1 hour at 37**°**C. ExoT was detected by anti-FLAG antibody.

**Supplemental Figure 2**


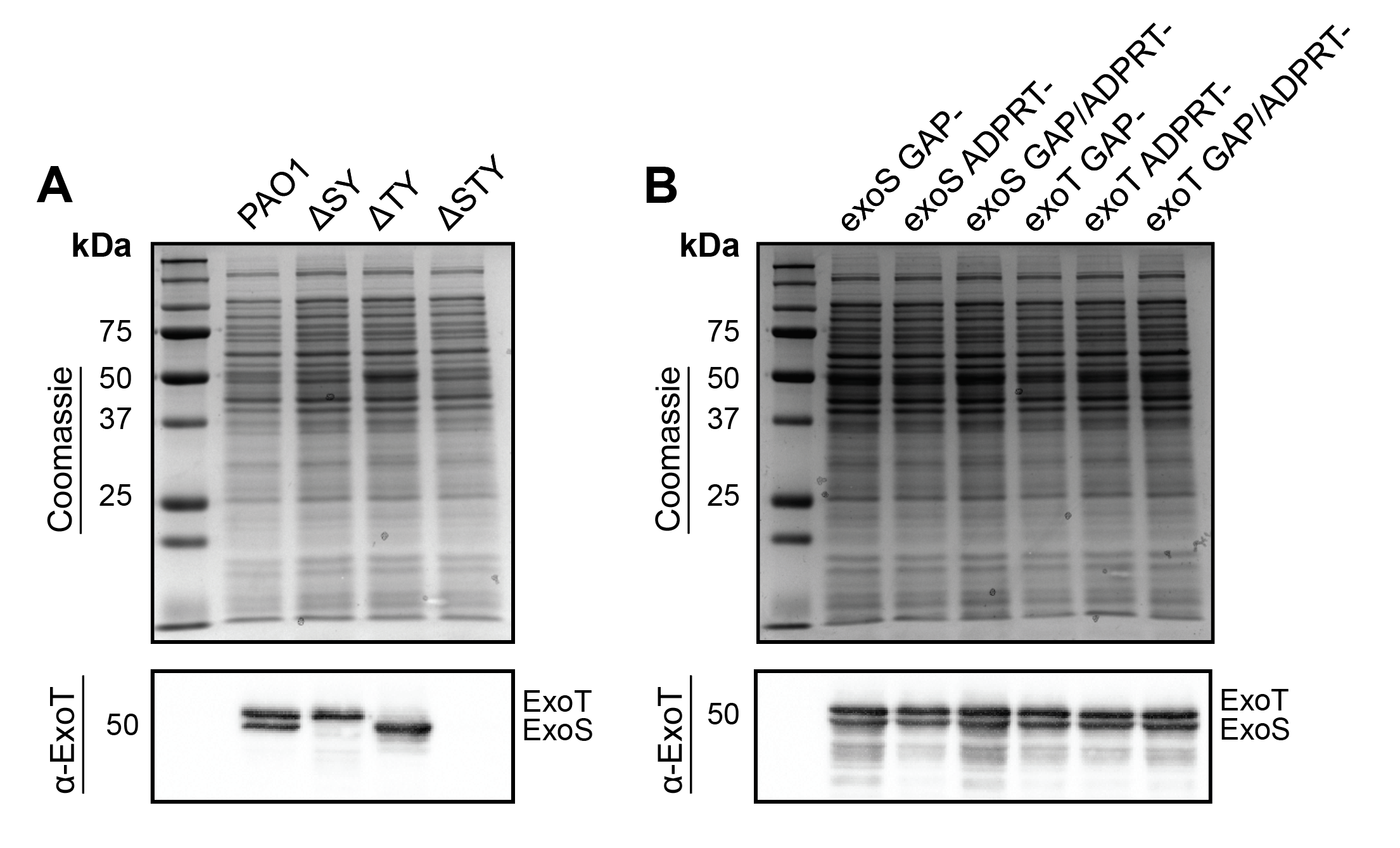


**Figure S2. Type-three secreted exotoxins by catalytic null mutant strains.** (**A**) WT PAO1 and exotoxin deletion mutants or (**B**) catalytic null mutants in PAO1 were grown in TSB with 100 mM monosodium glutamate and 1% glycerol supplemented with 2 mM EGTA to induce type-three exotoxin secretion for 6 hours. Secreted proteins were precipitated with trichloroacetic acid and loading volumes were normalized based on culture OD_540_ values prior to separation on a 12% polyacrylamide gel. Western blots were performed using rabbit α-ExoT antisera that cross-reacts with ExoS due to high similarity (76% identity) (36).

**Supplemental Figure 3**


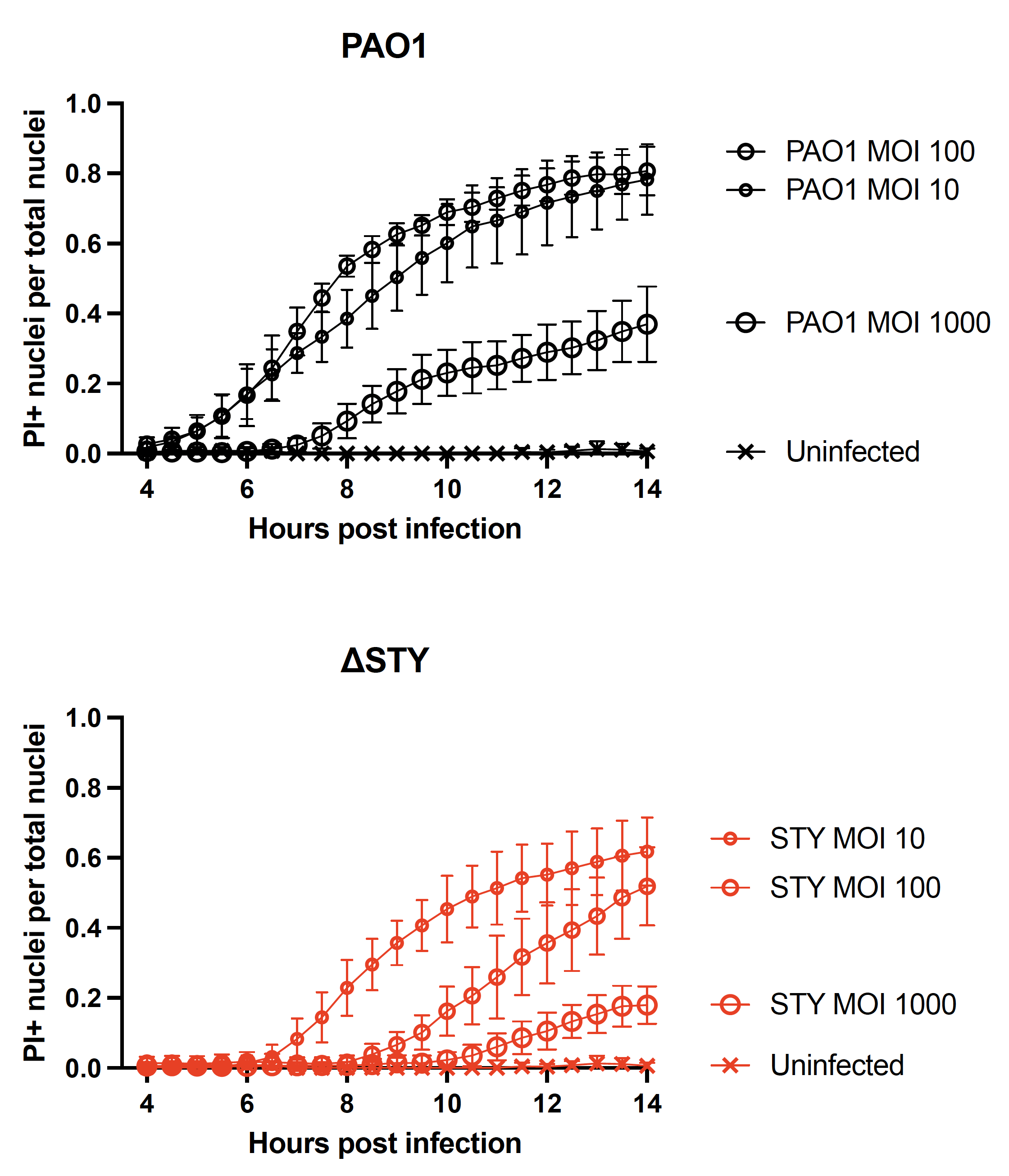


**Figure S3. Increased MOI reduces host cell death rates.** HeLa cells were labeled with Hoechst prior to infection. Cells were infected with PAO1 or PAO1∆STY at a MOI of 10, 100, or 1000 for 3 hours, treated with amikacin (0.2 mg/mL) and polymyxin B (10 μg/mL), and propidium iodide. Cells were imaged every 30 minutes for 14 hours. The ratio of propidium iodide-positive nuclei to total nuclei was measured in each frame and plotted as a percentage over time.

**Supplemental Table 1. Mutations detected following iterative allelic exchange.** Mutants with four T3SS exotoxin catalytic activities mutated in strain PAO1F were subjected to whole genome sequencing and compared for off-target mutations against the parent PAO1F strain. The strain and name of the deposited sequencing file is listed in the first column with each identified mutation detailed below. FastQ files were submitted to the NCBI Sequence Read Archive which can be accessed at the BioProject database under ID PRJNA1423707: http://www.ncbi.nlm.nih.gov/bioproject/1423707.

| **Mutation Class found each Strain** | **Position** | **Mutation** | **AA substitution** | **Gene** | **Description** |
| --- | --- | --- | --- | --- | --- |
| **exoS (R146K, E379D, E381D) exoT (R149K) exoY (K81M), 2_SG_A_TG** |  |  |  |  |  |
| target mutations | 59,230 | CG→AA | R149K (CGA→AAA) | exoT | exoenzyme T |
| target mutations | 2,411,239 | T→A | K81M (AAG→ATG) | exoY | adenylate cyclase |
| target mutations | 4,303,342 | C→T | E387E (GAG→GAA) | exoS | exoenzyme S |
| target mutations | 4,303,360 | C→A | E381D (GAG→GAT) | exoS | exoenzyme S |
| target mutations | 4,303,366 | T→A | E379D (GAA→GAT) | exoS | exoenzyme S |
| target mutations | 4,304,065 | CGT→AAA | R146K (CGT→AAA) | exoS | exoenzyme S |
| offsite mutations | 792,119 | C→T | D250D (GAC→GAT) | PA0724 | phage coat protein A |
| offsite mutations | 792,124 | G→A | G252D (GGC→GAC) | PA0724 | phage coat protein A |
| offsite mutations | 793,950 | T→C | L318L (CTT→CTC) | PA0726 | hypothetical protein |
|  |  |  |  |  |  |
| **exoS (R146K, E379D, E381D) exoT (E383D, E385D) exoY (K81M), 3_SG_A_TA** |  |  |  |  |  |
| target mutations | 59,934 | G→T | E383D (GAG→GAT) | exoT | exoenzyme T |
| target mutations | 59,940 | G→T | E385D (GAG→GAT) | exoT | exoenzyme T |
| target mutations | 2,411,239 | T→A | K81M (AAG→ATG) | exoY | adenylate cyclase |
| target mutations | 4,303,342 | C→T | E387E (GAG→GAA) | exoS | exoenzyme S |
| target mutations | 4,303,360 | C→A | E381D (GAG→GAT) | exoS | exoenzyme S |
| target mutations | 4,303,366 | T→A | E379D (GAA→GAT) | exoS | exoenzyme S |
| target mutations | 4,304,065 | CGT→AAA | R146K (CGT→AAA) | exoS | exoenzyme S |
| offsite mutations | 892,049 | A→G | I114T (ATA→ACA) | PA0813 | hypothetical protein |
|  |  |  |  |  |  |
| **exoS (R146K) exoT (R149K, E383D, E385D) exoY (K81M), 4_SG_TG_A** |  |  |  |  |  |
| target mutations | 59,230 | CG→AA | R149K (CGA→AAA) | exoT | exoenzyme T |
| target mutations | 59,934 | G→T | E383D (GAG→GAT) | exoT | exoenzyme T |
| target mutations | 59,940 | G→T | E385D (GAG→GAT) | exoT | exoenzyme T |
| target mutations | 2,411,239 | T→A | K81M (AAG→ATG) | exoY | adenylate cyclase |
| target mutations | 4,304,065 | CGT→AAA | R146K (CGT→AAA) | exoS | exoenzyme S |
|  |  |  |  |  |  |
| **exoS (E379D, E381D) exoT (R149K, E383D, E385D) exoY (K81M), 5_SA_TG_A** |  |  |  |  |  |
| target mutations | 59,230 | CG→AA | R149K (CGA→AAA) | exoT | exoenzyme T |
| target mutations | 59,934 | G→T | E383D (GAG→GAT) | exoT | exoenzyme T |
| target mutations | 59,940 | G→T | E385D (GAG→GAT) | exoT | exoenzyme T |
| target mutations | 2,411,239 | T→A | K81M (AAG→ATG) | exoY | adenylate cyclase |
| target mutations | 4,303,342 | C→T | E387E (GAG→GAA) | exoS | exoenzyme S |
| target mutations | 4,303,360 | C→A | E381D (GAG→GAT) | exoS | exoenzyme S |
| target mutations | 4,303,366 | T→A | E379D (GAA→GAT) | exoS | exoenzyme S |
| offsite mutations | 1,667,652 | A→C | D543A (GAC→GCC) | lig | NAD‑dependent DNA ligase LigA |

**Figure legends for supplemental movies**

**Movie S1. HeLa cell infection with *P. aeruginosa* T3SS mutants.** HeLa cells were infected with WT PAO1, a strain lacking exotoxins S, T, and Y (ΔSTY), or a T3SS-deficient mutant (Δ*exsA*) at an MOI of 10 for 3 hours. At 3 hpi, amikacin (0.2 mg/mL) and polymyxin B (10 μg/mL) were supplemented and cells were imaged every 30 minutes for 10 hours.

**Movie S2. HeLa cell infection with *P. aeruginosa* expressing only ExoS functional domains.** HeLa cells were infected with a *P. aeruginosa* strain expressing only ExoS (ΔTY), or versions of ExoS where only the GAP or ADPRT domains are catalytically active at an MOI of 10 for 3 hours. At 3 hpi, amikacin (0.2 mg/mL) and polymyxin B (10 μg/mL) were supplemented and cells were imaged every 30 minutes for 10 hours.

**Movie S3. HeLa cell infection with *P. aeruginosa* expressing only ExoT functional domains.** HeLa cells were infected with a *P. aeruginosa* strain expressing only ExoT (ΔSY), or versions of ExoT where only the GAP or ADPRT domains are catalytically active at an MOI of 10 for 3 hours. At 3 hpi, amikacin (0.2 mg/mL) and polymyxin B (10 μg/mL) were supplemented and cells were imaged every 30 minutes for 10 hours.

**Movie S4. HeLa cell infection with *P. aeruginosa* ExoS catalytic null mutants.** HeLa cells were infected with a *P. aeruginosa* strain where only the GAP and/or ADPRT domains of ExoS are catalytically inactivated at an MOI of 10 for 3 hours. At 3 hpi, amikacin (0.2 mg/mL) and polymyxin B (10 μg/mL) were supplemented and cells were imaged every 30 minutes for 10 hours.

**Movie S5. HeLa cell infection with *P. aeruginosa* ExoT catalytic null mutants.** HeLa cells were infected with a *P. aeruginosa* strain where only the GAP and/or ADPRT domains of ExoT are catalytically inactivated at an MOI of 10 for 3 hours. At 3 hpi, amikacin (0.2 mg/mL) and polymyxin B (10 μg/mL) were supplemented and cells were imaged every 30 minutes for 10 hours.

**Movie S6. HeLa cell infection with *P. aeruginosa* ExoS and ExoT combination catalytic null mutants.** HeLa cells were infected with a *P. aeruginosa* strain where a single functional domain of both ExoS and ExoT are catalytically inactivated at an MOI of 10 for 3 hours. At 3 hpi, amikacin (0.2 mg/mL) and polymyxin B (10 μg/mL) were supplemented and cells were imaged every 30 minutes for 10 hours.

**Movie S7. Increasing the MOI represses *P. aeruginosa* T3SS activation.** HeLa cells were infected with WT PAO1 at MOI 10, 100, or 1000 for 3 hours. At 3 hpi, cells were treated with amikacin (0.2 mg/mL) and polymyxin B (10 μg/mL) and imaged every 30 minutes for 14 hours.

**Movie S8. Extracellular T3SS delivery of ExoS ADPRT promotes intracellular vacuolar escape.** HeLa cells were infected with an ExoS GAP^+^-expressing strain (upper panels), or with a ∆*exsA* mutant (lower panels), each transformed with pCG-P_exoS_-mS, at an MOI 1000 for 2 hours. At 2 hpi, cells were treated with amikacin (0.2 mg/mL) and polymyxin B (10 μg/mL) for 1 hour, and then subsequently infected with indicated non-fluorescent mutant strain expressing a single active ExoS or ExoT domain at a MOI of 10 for 3 hours. Infected cells were treated again with antibiotics to eliminate extracellular bacteria and imaged every 30 minutes for 14 hours.

**Movie S9. Primary human corneal epithelial cell infection with *P. aeruginosa* T3SS mutants.** Corneal cells were infected with WT PAO1, a strain lacking exotoxins S, T, and Y (ΔSTY), or a T3SS-deficient mutant (Δ*exsA*) at an MOI of 10 for 3 hours. In the lower panels, corneal cells were infected with strains where only indicated single catalytic activities from either ExoS or ExoT were present. At 3 hpi, amikacin (0.2 mg/mL) and polymyxin B (10 μg/mL) were supplemented and cells were imaged every 30 minutes for 10 hours.
